# TAB2 Causes Neuronal Damage by Aggravating Microglia‐Mediated Neuroinflammation in Parkinson's Disease

**DOI:** 10.1002/advs.77175

**Published:** 2026-08-17

**Authors:** Yanhao Zhao, Zimeng Huang, Jianing Gao, Aolan Li, Huizhi Wang, Yingchuan Chen, Guanyu Zhu, Xin Zhang, Yin Jiang, Moxuan Zhang, Fangang Meng, Jianguo Zhang, Tingting Du

**Affiliations:** ^1^ Department of Functional Neurosurgery Beijing Neurosurgical Institute Capital Medical University Beijing China; ^2^ Department of Neurosurgery Beijing Tiantan Hospital Capital Medical University Beijing China; ^3^ Department of Neurosurgery Shandong Cancer Hospital and Institute Shandong First Medical University and Shandong Academy of Medical Sciences Jinan China

**Keywords:** lumacaftor, neuroinflammation, parkinson's disease, TAB2

## Abstract

Neuroinflammation plays a key role in exacerbating dopaminergic neuron loss in Parkinson's disease (PD). We identified TAB2 as an early‐stage biomarker, which was elevated in PD patients’ microglia. However, the role of TAB2 in the pathogenesis of PD remains unknown. In this study, we found that *Tab2* knockdown inhibited the activation of microglia and protected neurons in PD models. STAT3, as a transcription factor for TAB2, regulated TAB2 expression. Mechanistically, TAB2 interacted with α‐synuclein and facilitated the recognition of K63‐linked ubiquitin chains, leading to the formation of the TAK1–TABs complex and activation of TAK1, which was ultimately followed by activation of the nuclear factor‐kappa B (NF‐κB) signaling pathway. Furthermore, microglia‐specific knockdown of *Tab2* significantly inhibited microglia activation, protected dopaminergic neurons, improved motor function, and attenuated anxiety‐like behaviors in PD mouse model. We further showed that the FDA‐approved drug, lumacaftor, suppressed microglial TAB2 expression and had potent anti‐inflammatory and neuroprotective effects in PD models. Taken together, our study reveals that the STAT3–TAB2–NF‐κB–IL‐1β positive feedback axis in microglia is a crucial checkpoint that exacerbates neuroinflammation in PD. Therefore, these findings identify a pivotal role of TAB2 in regulating microglia‐mediated neuroinflammation, suggesting that targeting TAB2 may be a possible therapeutic strategy for PD.

## Graphical abstract

In microglia, STAT3 upregulates TAB2, which promotes NF‐κB activation through its NZF domain‐mediated recognition of K63‐linked ubiquitin chains, leading to inflammatory cytokine release and subsequent neuronal injury. Lumacaftor suppresses TAB2 expression and directly binds the TAB2‐NZF domain to interrupt K63 ubiquitin recognition, thereby blocking NF‐κB signaling and neuroinflammation to exert neuroprotective effects.

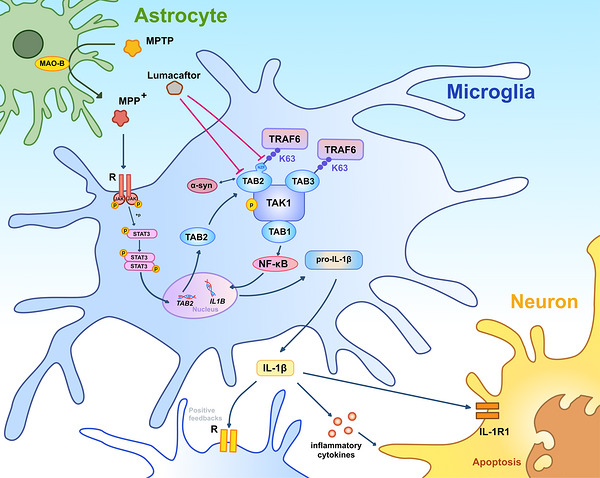

## 1 Introduction

Parkinson's disease (PD) is a neurodegenerative disorder characterized by progressive degeneration of midbrain substantia nigra dopaminergic neurons, affecting 1%–2% of individuals over 65 years of age and 4% over 80 years of age [1]. As global population aging accelerates, PD incidence continues to rise, posing a major socioeconomic burden. While the exact etiology remains elusive, interactions among genetic, environmental, and aging factors have been implicated [2]. Current dopamine replacement therapy is only effective in the early stages and causes severe side effects with long‐term use, emphasizing the urgent need to elucidate PD pathogenesis and develop novel therapies.

Microglia‐mediated neuroinflammation is significantly elevated in PD, Alzheimer's disease, and various other neurodegenerative disorders [3, 4]. Recent evidence demonstrates that neuroinflammation is not merely a consequence of neuronal death, but a key driver of PD progression [3]. The α‐synuclein (α‐Syn), a pathological hallmark of PD, is emerging as a component bridging neuro/immune‐inflammatory responses and neurodegeneration in PD patients [5], acting as both a pathogenic factor and a damage‑associated molecular pattern that activates microglia, which in turn propagate α‑Syn to healthy brain regions [6]. Critically, neuronal loss occurs before Lewy body formation [7], and microglial activation precedes dopaminergic neuron death in animal models [8, 9], establishing neuroinflammation as an early and central event in PD.

TAB2 (TAK1‐binding protein 2) and its homolog TAB3 are essential components of the TAK1–TABs complex [10]. Upon inflammatory stimulation by IL‐1β, TNF‐α, or lipopolysaccharide (LPS), TAB2/3 recognizes K63‐linked ubiquitin chains via their Npl4 Zinc Finger (NZF) domains, recruiting TAK1–TAB1 to form an active tetramer that drives TAK1 autophosphorylation and subsequent activation of Mitogen‐activated protein kinase (MAPK) and nuclear factor‐kappa B (NF‐κB) cascades [11]. TAB2 has been implicated in inflammatory bowel disease pathogenesis. In Crohn's disease, LRRK2 interacts with TAB2 to activate the NF‐κB pathway, concurrent with promoting Beclin‐1 degradation and autophagy inhibition [12]. In models of colitis, RNF99 targets TAB2 for K48‐linked ubiquitination and degradation, thereby attenuating NF‐κB‐ and MAPK‐mediated intestinal inflammation and conferring protective effects [13]. Within the central nervous system, TAB2 serves as an inflammatory amplifier in ischemic stroke, where USP25 suppresses post‐ischemic neuroinflammatory responses through TAB2 deubiquitination [14]. Collectively, these findings emphasize the pivotal role of TAB2 in NF‐κB‐driven inflammatory cascades. Because NF‐κB is a critical driver of neuroinflammation, which is persistently activated in the PD midbrain until end‐stage disease [15], the TAK1–TABs complex represents a key therapeutic target.

In the present study, we identified the regulatory mechanism of TAB2 in the neuroinflammation of PD. Our results showed that a STAT3–TAB2–NF‐κB–IL‐1β positive feedback axis in microglia exacerbated PD neuroinflammation. *Tab2* knockdown/lumacaftor treatment exerted neuroprotective effects by inhibiting TAB2 protein expression and directly binding to the TAB2‐NZF domain to block TAB2‐mediated recognition of K63‐linked ubiquitin chains and inhibit TAK1 phosphorylation, thereby interrupting the NF‐κB signaling pathway, suggesting that targeting TAB2 represented a promising therapeutic strategy for PD.

## 2 Results

### 2.1 Single‐Nucleus RNA Sequencing (snRNA‐seq) Revealed Microglial Heterogeneity and Identified *TAB2* as a Critical Gene in PD

**FIGURE 1:**
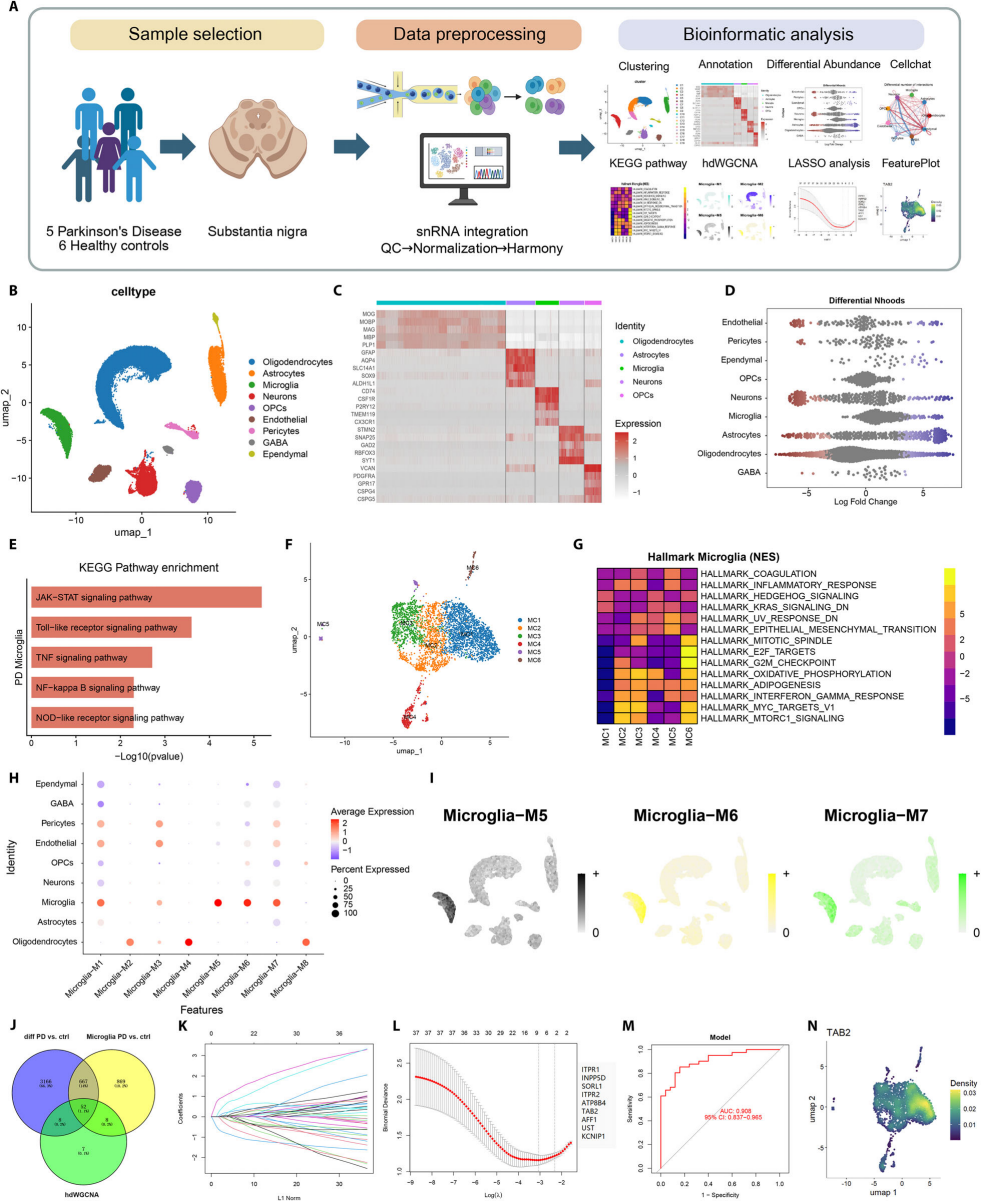
Molecular heterogeneity analysis of microglial cells. (A) Schematic overview of the study design and analysis workflow. (B) The UMAP plot showed the single‐nucleus clustering data and subpopulation annotations from six HCs and five PD patients in the substantia nigra. (C) Heat map showing representative marker genes across the five major cell types. (D) Beeswarm plot showing the cell type abundance between PD patients and HCs in the substantia nigra. (E) The Kyoto Encyclopedia of Genes and Genomes enrichment analyses of differentially expressed pathways in PD microglia versus HCs. (F) The UMAP plot showed the projections of microglia and displayed by cluster number. (G) The heat map shows the GSEA Hallmark Pathway gene set for each subclusters. (H) High‐dimensional weighted gene co‐expression network analyses module activities across major cell types are shown. (I) Feature plots showed the spatial distribution of representative microglia‐activated modules (M5, M6, and M7) on the UMAP. (J) Venn diagram showed the overlap of candidate genes. (K, L) LASSO regression for feature selection in bulk transcriptomic data, including coefficient profiles and cross‐validation to determine the optimal penalty parameter λ are shown. (M) Receiver Operating Characteristic curve analysis evaluated the predictive performance of the LASSO‐selected gene signature. (N) The UMAP feature plot shows the distribution of *TAB2* levels within microglia.

The data analysis process is shown in Figure 1A. We analyzed the midbrain snRNA‐seq data from five PD patients and six healthy controls (HCs). Using quality controls, we retained 40 472 high‐quality nuclei from these midbrains for further analysis. Following the Seurat preprocessing pipeline [including normalization, principal component analysis (PCA), and Harmony‐based integration], we visualized the clusters using a Uniform Manifold Approximation and Projection (UMAP) plot to identify 19 distinct clusters (C1–C19) (Figure S1A). We also showed the distribution of individual cells in the UMAP plot for each patient (Figure S1B). By manually comparing cluster‐specific marker genes with well‐known markers reported in the literature, we annotated nine distinct cell types (Figure 1B).

The heat map displays the top expressed genes across the five major cell types (Figure 1C). Among these, oligodendrocytes were characterized by high expression of MOBP and MOG, astrocytes predominantly expressed GFAP and AQP4, and microglia were marked by TMEM119 and CX3CR1 expression (Figure S1C). Given the low abundance of dopaminergic neurons (0.14% of all cells), we did not annotate them as a separate cell type at this stage. We also determined the distribution of cell types in each patient (Figure S1D). Differential abundance analysis revealed a marked reduction in neurons in PD patients, accompanied by an expansion of microglia (Figure 1D). Consistently, the box plot showed a significantly higher microglial abundance in PD patients, when compared with controls (*p* = 0.0087) (Figure S1E). Given these results, we selected microglia as the primary cell type for subsequent in‐depth analyses. Functional enrichment analysis of microglia highlighted a prominent immune‐activated and pro‐inflammatory signature in PD. In the Gene Ontology (GO) category, PD microglia were primarily enriched in cell activation involved in immune response, canonical NF‐κB signal transduction, regulation of interleukin‐6 production, neuroinflammatory response, and microglial cell activation (Figure S1F). Consistently, Kyoto Encyclopedia of Genes and Genomes (KEGG) analysis showed significant enrichment of inflammation‐related pathways, including the JAK‐STAT signaling pathway, Toll‐like receptor signaling pathway, TNF signaling pathway, NF‐κB signaling pathway, and NOD‐like receptor signaling pathway (Figure 1E). Cell communication analysis showed that microglia enhanced communication with multiple cell types (including oligodendrocytes and neurons) in PD patients (Figure S2A,B). Taken together, these results indicated that microglia in PD exhibited heightened inflammatory activation and immune signaling remodeling.

To further determine the function of microglia in PD, we performed sub‐clustering of microglia. We identified a total of six microglia subclusters (Figure 1F, Figure S2C,D). Among them, MC1‐MC3 are the three largest groups of microglia. To explore the dynamic relationships among these subpopulations, we further conducted trajectory analysis. The results revealed a continuous phenotypic trajectory originating from MC1 and progressing toward MC2 and MC3 (Figure S2E). We then generated a dot plot to display the top differentially expressed genes across microglial clusters (Figure S2F). The MC1 subset showed high expression of *P2RY12*, a marker typically enriched in resting microglia. In contrast, MC2 and MC3 exhibited elevated *HSP90AA1* and *GPNMB*, respectively, both of which were associated with activated microglia. In Figure 1G, hallmark gene set enrichment analysis data revealed marked functional heterogeneity across microglial subclusters. Notably, MC2 and MC3 exhibited an inflammatory bias, showing prominent enrichment for inflammatory response, oxidative phosphorylation, and interferon gamma response. In contrast, the other subclusters displayed relatively non‐inflammatory functional features. Overall, these enrichment patterns indicate that MC2 and MC3 represented the major inflammation‐associated microglial subpopulations in PD. We used high‐dimensional weighted gene co‐expression network analysis (hdWGCNA) to identify key microglial molecular modules. During network construction, we selected a soft‐thresholding power (β) = 4, at which the scale‐free topology fit index reached 0.85, yielding optimal network connectivity (Figure S3A). We identified a total of eight gene modules (Figure S3B,C). We then evaluated the expression characteristics of each module and found that the M5, M6, and M7 modules were highly activated in microglia (Figure 1H,I).

To further screen core genes, we conducted differential analysis and identified a total of 52 genes (Figure 1J). Among the 52 candidate genes, 37 genes were retained for least absolute shrinkage and selection operator (LASSO) analysis after matching gene symbols to the bulk transcriptomic dataset and removing genes that were absent, duplicated, or of low expression in the bulk expression matrix. Subsequently, we used LASSO for the bulk transcriptomic data for feature selection and identified nine candidate genes (Figure 1K,L). We then built a regression‐based predictive model using these genes, which demonstrated good predictive performance (Figure 1M). Box plot results showed that *AFF1*, *SORL1*, *INPP5D*, and *TAB2* were significantly upregulated in the substantia nigra of PD patients (Figure S3D–G). Among these, we prioritized *TAB2* for downstream analyses given its well‐established role in inflammatory signaling. Based on snRNA‐seq data, *TAB2* level was predominantly enriched in the MC1 microglial subcluster (Figure 1N). Furthermore, *TAB2* expression was significantly elevated in both MC1 and MC2 microglia from PD patients compared with controls, whereas no significant difference was observed in MC3 (Figure S2G). Notably, *IL1B* was primarily enriched in the MC2 subcluster, which also exhibited elevated *HSP90AA1* and enrichment of inflammatory response pathways, indicating a pronounced inflammatory phenotype. Together, these findings suggested that *TAB2* upregulation may occur at an early stage of microglial activation and contribute to the initiation and amplification of inflammatory cascades during PD pathogenesis.

### 2.2 Elevated TAB2 Expression was a Conserved Feature Across Multiple PD Models, and Correlated With NF‐κB Pathway Activation

**FIGURE 2:**
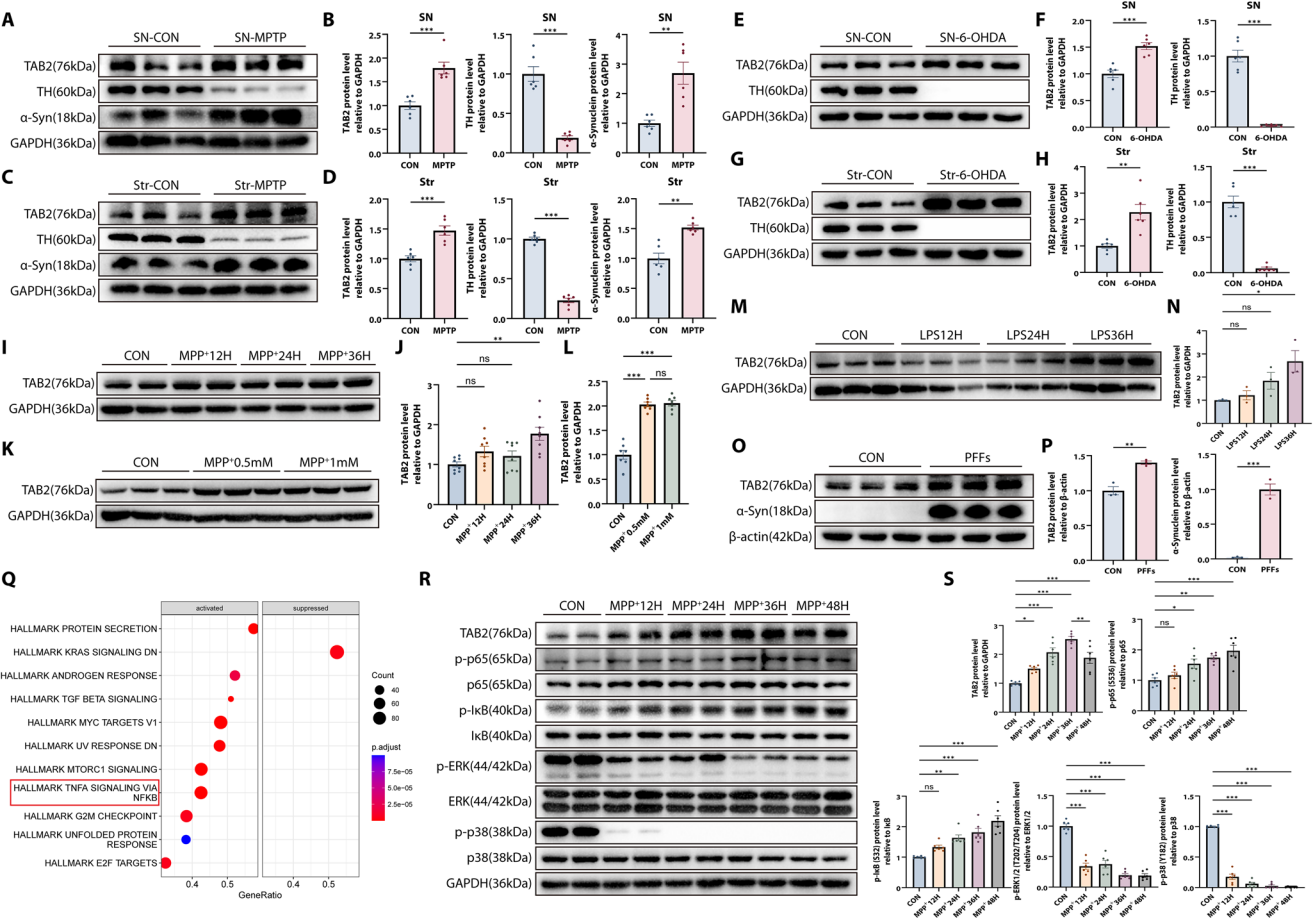
The TAB2 expression was elevated in multiple PD models and was related to the NF‐κB pathway. (A, B) TAB2, TH, and α‐Syn expression in the substantia nigra of MPTP‐induced PD mouse models (*n* = 6). (C, D) TAB2, TH, and α‐Syn expression in the striatum of MPTP‐induced PD mouse models (*n* = 6). (E, F) TAB2 and TH expression in the substantia nigra of 6‐OHDA‐induced PD rat models (*n* = 6). (G, H) TAB2 and TH expression in the striatum of 6‐OHDA‐induced PD rat models (*n* = 6). (I, J) Time‐dependent TAB2 expression in BV‐2 cells treated with 0.5 mm MPP^+^ for 12 h, 24 h, and 36 h (*n* = 8). (K, L) Dose‐dependent TAB2 expression in BV‐2 cells treated with 0.5 and 1 mm MPP^+^ for 36 h (*n* = 7). (M, N) Time‐dependent TAB2 expression in BV‐2 cells treated with 1µg/mL lipopolysaccharide for 12, 24, and 36 h (*n* = 3). (O, P) TAB2 and α‐Syn expression in BV‐2 cells treated with 1µg/mL α‐Syn PFFs for 36 h (*n* = 3). (Q) GSEA analyses of PD snRNA‐seq datasets revealed significant activation of inflammatory pathways, including the NF‐κB signaling axis (highlighted in red). (R, S) Expression of TAB2, NF‐κB pathway (p‐p65, p65, p‐IκB, and IκB), and MAPK pathway (p‐ERK1/2, ERK1/2, p‐p38, and p38) proteins in BV‐2 cells treated with 0.5 mm MPP^+^ for 12, 24, 36, and 48 h (*n* = 6). Quantitative data are presented as the mean ± SEM. ^*^ *p* < 0.05, ^**^ *p* < 0.01, and ^***^ *p* < 0.001.

To determine whether TAB2 elevation was conserved in PD models, we examined the expression of TAB2 in the substantia nigra and striatum of 1‐methyl‐4‐phenyl‐1,2,3,6‐tetrahydropyridine (MPTP)‐treated mice. Western blot analysis revealed increased TAB2 expression, decreased TH levels, and elevated α‐Syn in both regions (Figure 2A–D). Similarly, in 6‐hydroxydopamine (6‐OHDA)‐induced rat models, TAB2 expression was upregulated, while TH expression was downregulated in the substantia nigra and striatum (Figure 2E–H).

We next established PD neuroinflammation models using BV‐2 microglial cells. 1‐methyl‐4‐phenylpyridinium (MPP^+^) treatment induced a time‐dependent increase in TAB2, with an elevation at 36 h. However, increasing the MPP^+^ concentration did not further elevate TAB2 levels, indicating that this effect was not concentration‐dependent (Figure 2I–L). Therefore, 0.5 mm MPP^+^ treatment for 36 h was used as the optimal condition for subsequent experiments. Similarly, in LPS‐treated cells, TAB2 levels increased in a time‐dependent manner, with significant elevation at 36 h (Figure 2M, N). The α‐Syn preformed fibrils (PFFs) treatment significantly increased α‐Syn levels, confirming successful model establishment and elevated TAB2 expression (Figure 2O, P).

Analysis of snRNA sequencing data revealed that inflammatory pathways, particularly the NF‐κB pathway, were enriched in microglia from PD patients (Figure 2Q). We therefore examined NF‐κB and MAPK signaling pathways in MPP^+^‐treated BV‐2 cells. Importantly, TAB2 protein levels peaked at 36 h, showed a decreasing tendency at 48 h, but remained higher than the control group, whereas phosphorylated p65 and IκB levels increased progressively over time. In contrast, MAPK pathway activity (phosphorylated ERK and p38) significantly decreased with prolonged treatment, and exhibited no transient activation even at early time points (5, 15, and 30 min; 1, 2, 4, and 6 h), indicating that inflammatory responses in MPP^+^‐treated BV‐2 cells were primarily mediated by the NF‐κB pathway (Figure 2R,S; Figure S4). Together, these results suggested that TAB2 expression was elevated in multiple PD models and may be related to the NF‐κB pathway, but not the MAPK pathway.

### 2.3 Tab2 Knockdown Inhibited Neuroinflammation and Exerted Neuroprotective Effects

**FIGURE 3:**
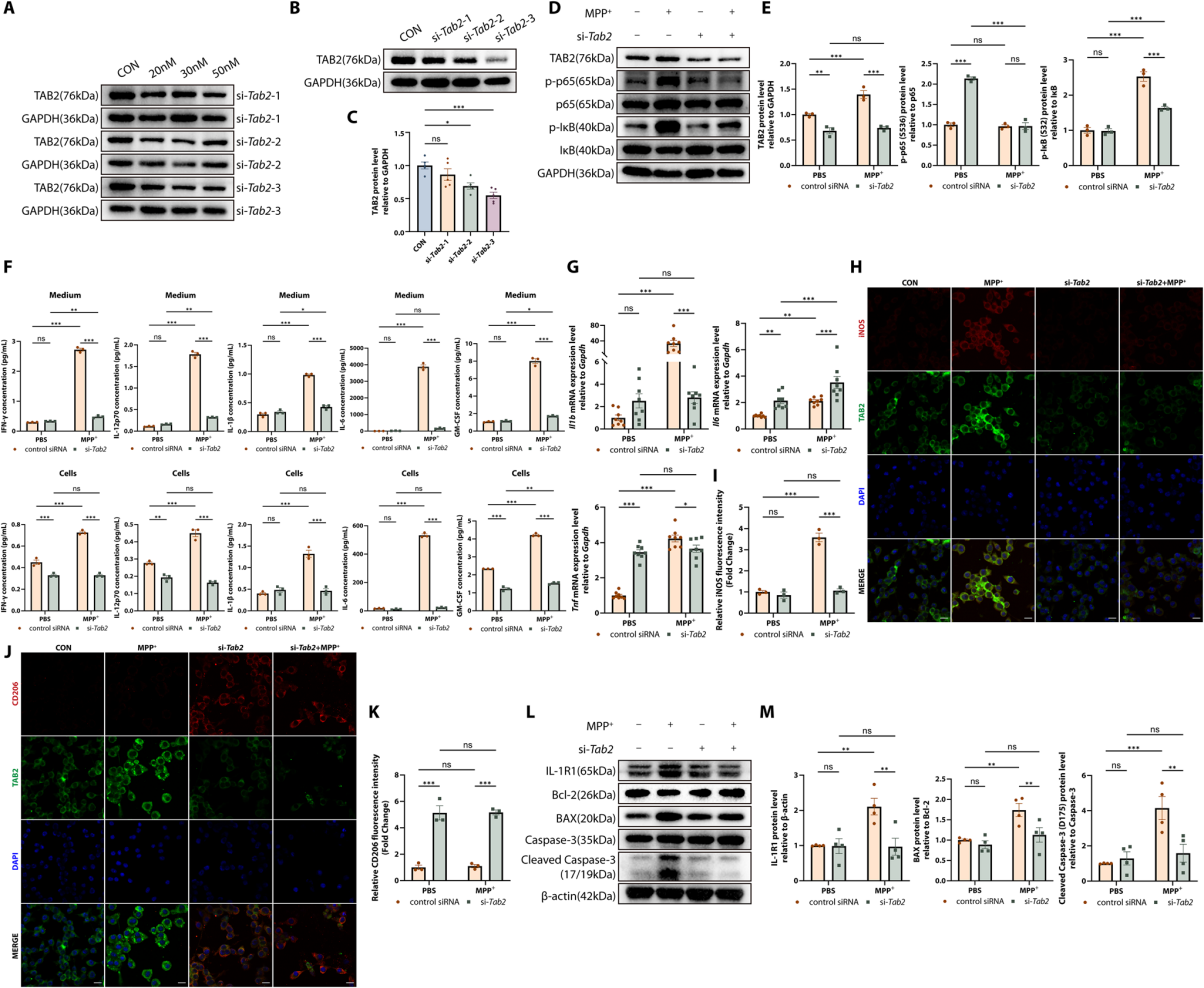
*Tab2* knockdown promoted microglial polarization toward an anti‐inflammatory phenotype and exerted neuroprotective effects. (A–C) Screening of three siRNA sequences targeting *Tab*2 to identify the most effective knockdown and optimal concentration in BV‐2 cells (*n* = 5). (D, E) Expression of TAB2 and the NF‐κB pathway (p‐p65, p65, p‐IκB, and IκB) proteins in BV‐2 cells transfected with control siRNA or *Tab2* siRNA (30 nm, 42 h), followed by treatment with or without MPP^+^ (*n* = 3). (F) Cytokine levels in BV‐2 cell lysates and culture supernatants were measured using the Luminex assay (*n* = 3). (G) The mRNA expression of *Il1b*, *Il6*, and *Tnf* in BV‐2 cells transfected with control siRNA or *Tab2* siRNA, followed by treatment with or without MPP^+^, was measured by RT‐qPCR (*n* = 8). (H–I) Immunofluorescence staining of TAB2 and iNOS expression in BV‐2 cells transfected with control siRNA or *Tab2* siRNA, followed by treatment with or without MPP^+^ (*n* = 3) (Scale bar, 20 µm). (J–K) Immunofluorescence staining of TAB2 and CD206 expression in BV‐2 cells transfected with control siRNA or *Tab2* siRNA, followed by treatment with or without MPP^+^ (*n* = 3) (Scale bar, 20 µm). (L, M) Expression of IL‐1R1 and apoptosis pathway components (Bcl‐2, BAX, Caspase‐3, and cleaved Caspase‐3) in SH‐SY5Y cells co‐cultured with BV‐2 cells in a Transwell system. BV‐2 cells (upper chamber) were transfected with control siRNA or *Tab2* siRNA and treated with or without MPP^+^ (*n* = 4). Quantitative data are presented as the mean ± SEM. ^*^ *p* < 0.05, ^**^ *p* < 0.01, and ^***^ *p* < 0.001.

We designed three siRNAs targeting *Tab2*, and optimized the knockdown efficiency in BV‐2 cells. The 30 nm si‐*Tab2*‐3 resulted in the most effective TAB2 depletion (Figure 3A–C). Cells were then divided into four groups: (1) control siRNA + PBS, (2) control siRNA + MPP^+^, (3) si‐*Tab2* + PBS, and (4) si‐*Tab2* + MPP^+^. Western blot analysis revealed that MPP^+^ treatment activated the NF‐κB pathway, when compared with the control group, whereas *Tab2* knockdown significantly attenuated NF‐κB activation in the si‐*Tab2* + MPP^+^ group (Figure 3D,E).

We further analyzed the inflammatory cytokine profiles using the Luminex assay. Five cytokines (IFN‐γ, IL‐12p70, IL‐1β, IL‐6, and GM‐CSF) showed increased levels in both cell culture supernatants and cell lysates in the MPP^+^ group, which were reduced upon *Tab2* knockdown (Figure 3F). Real‐time quantitative polymerase chain reaction (RT‐qPCR) analysis further revealed that among IL‐1β, IL‐6, and TNF‐α, only IL‐1β transcription followed the same pattern of elevation by MPP^+^ and reduction by *Tab2* knockdown (Figure 3G). These results collectively indicated that TAB2 could regulate IL‐1β levels in the PD cell model.

Immunofluorescence staining was performed on Cell Climbing Tablet cells to determine the expression levels of the pro‐inflammatory marker, iNOS, and anti‐inflammatory marker, CD206. We found that MPP^+^ treatment significantly increased TAB2 and iNOS fluorescence intensity in BV‐2 cells, indicating a shift toward a pro‐inflammatory phenotype. *Tab2* knockdown reduced iNOS fluorescence intensity and significantly elevated CD206 fluorescence intensity, when compared with the MPP^+^ group, suggesting a transition to an anti‐inflammatory phenotype (Figure 3H–K). Together, these results suggested that *Tab2* knockdown inhibited neuroinflammation in the PD cell model.

We subsequently co‐cultured BV‐2 and SH‐SY5Y cells to evaluate the effect of neuroinflammation on a human neuroblastoma cell line (SH‐SY5Y cells) using Transwell inserts. Western blot analysis of SH‐SY5Y cells revealed that IL‐1R1 levels were significantly elevated in the MPP^+^ group, whereas *Tab2* knockdown markedly reduced IL‐1R1 levels. Apoptosis‐related proteins (BAX/Bcl‐2 and cleaved Caspase‐3/Caspase‐3) levels were significantly increased in the MPP^+^ group and decreased after *Tab2* knockdown (Figure 3L,M), confirming that *Tab2* knockdown in microglial cells resisted MPP^+^‐induced inflammatory responses and protected SH‐SY5Y cells against cell death.

### 2.4 STAT3 Transcriptionally Regulated TAB2 Expression and Modulated NF‐κB Pathway Activation In Vitro and In Vivo PD Models

**FIGURE 4:**
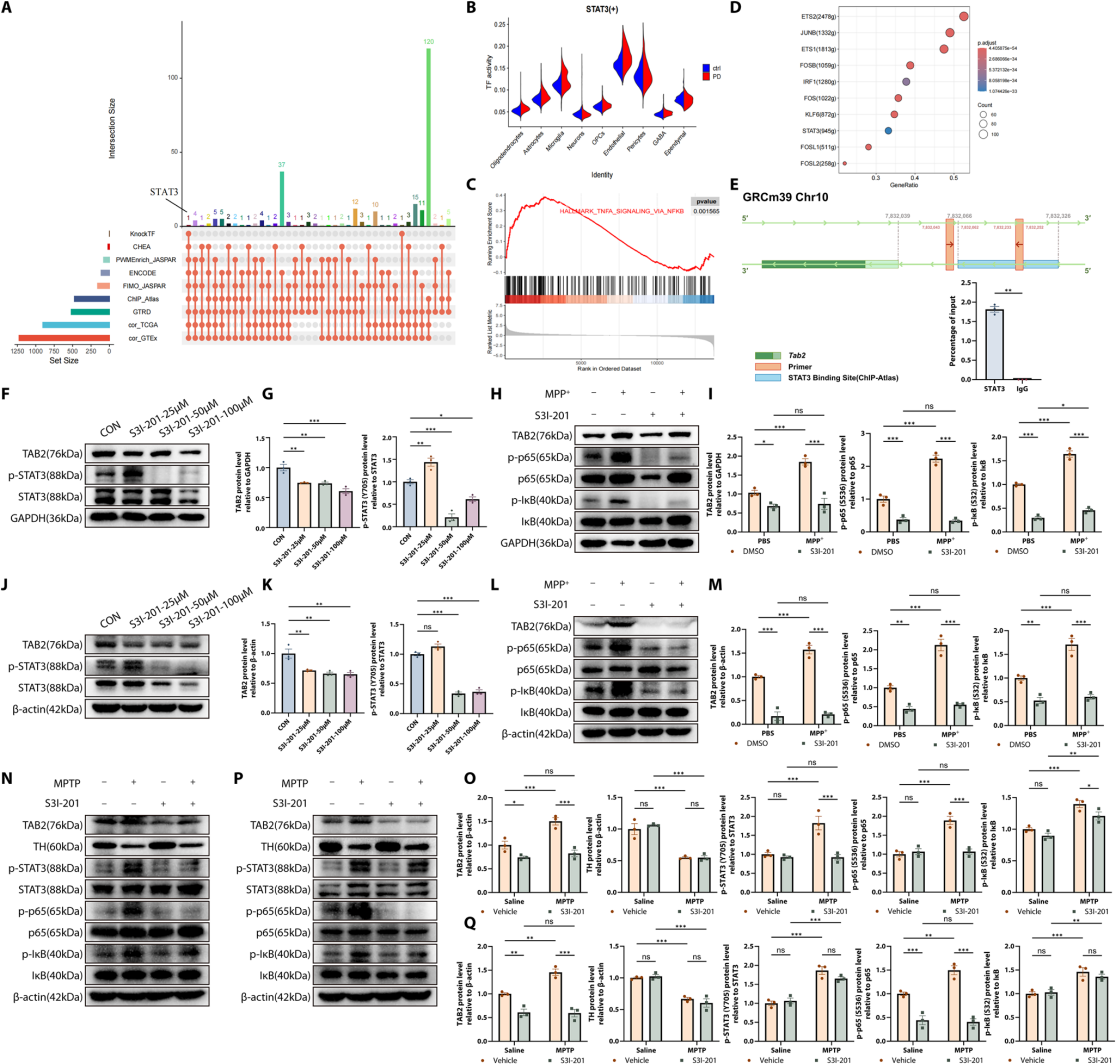
STAT3 regulated TAB2 expression and the NF‐κB signaling pathway. (A) Transcription factor prediction analyses identified STAT3 as a regulator of TAB2, which was performed using the TFTF R package (version 0.1.0). (B–D) SCENIC analysis identified STAT3 as a transcriptional regulator of TAB2 in PD patient midbrain microglia. (E) ChIP‐qPCR validation of STAT3 binding to the *Tab2* promoter. (F, G) Dose‐dependent effects of the STAT3 inhibitor, S3I‐201, on TAB2 expression and STAT3 phosphorylation in BV‐2 cells (*n* = 3). (H, I) Expression of TAB2 and NF‐κB pathway components (p‐p65, p65, p‐IκB, and IκB) in BV‐2 cells treated with dimethyl sulfoxide (DMSO) [0.5% (v/v), 1 h] or S3I‐201 (50 µm, 1 h), followed by treatment with or without MPP^+^ (0.5 mm, 36 h) (*n* = 3). (J, K) Dose‐dependent effects of the STAT3 inhibitor, S3I‐201, on TAB2 expression and STAT3 phosphorylation in primary microglia (*n* = 3). (L, M) Expression of TAB2 and NF‐κB pathway components (p‐p65, p65, p‐IκB, and IκB) in primary microglia treated with DMSO [0.5% (v/v), 1 h] or S3I‐201 (50 µm, 1 h), followed by treatment with or without MPP^+^ (0.5 mm, 36 h) (*n* = 3). (N–Q) Expression of TAB2, p‐STAT3, STAT3, TH, and NF‐κB pathway components (p‐p65, p65, p‐IκB, and IκB) in the substantia nigra (N, O) and striatum (P, Q) of mice treated with vehicle or S3I‐201 (10 mg/kg/d, i.p.), with or without MPTP administration (*n* = 3). Quantitative data are presented as the mean ± SEM. ^*^ *p* < 0.05, ^**^ *p* < 0.01, and ^***^ *p* < 0.001.

To identify transcription factors regulating TAB2, we used the integrated prediction framework of the TFTF R package, which consolidated evidence from multiple curated databases and algorithms, including KnockTF, CHEA, PWMEnrich_JASPAR, ENCODE, FIMO_JASPAR, ChIP_Atlas, and GTRD [16]. Correlation analysis using TCGA GBM data and GTEx brain tissue data revealed that nine datasets consistently supported STAT3 as a transcription factor for TAB2 (Figure 4A). We further analyzed single‐nucleus transcriptome data using SCENIC and found that STAT3 activity was significantly elevated in microglia from PD patients. GSEA analysis demonstrated activation of the NF‐κB pathway in PD patient microglia, with STAT3 target genes enriched within this pathway (Figure 4B–D) [17].

Subsequently, we performed chromatin immunoprecipitation followed by qPCR (ChIP‐qPCR) to validate STAT3 as a transcription factor for TAB2. ChIP‐qPCR analysis revealed that the TAB2 promoter was enriched in the STAT3 immunoprecipitate at approximately 1.8% of input, confirming that STAT3 served as a transcription factor for TAB2 (Figure 4E).

We then treated BV‐2 cells with the STAT3 inhibitor, S3I‐201. Testing various concentrations revealed that 50 µm achieved optimal p‐STAT3 inhibition and concurrently suppressed TAB2 expression (Figure 4F, G). Cells were divided into four groups: dimethyl sulfoxide (DMSO) + PBS, DMSO + MPP^+^, S3I‐201 + PBS, and S3I‐201 + MPP^+^. Similar to si‐*Tab2*, S3I‐201 treatment significantly decreased TAB2 protein levels while suppressing MPP^+^‐induced NF‐κB pathway activation (Figure 4H, I). To further validate these findings, we performed parallel experiments in primary microglia. Consistent with the results obtained in BV‐2 cells, S3I‐201 treatment significantly reduced TAB2 expression and suppressed MPP^+^‐induced NF‐κB pathway activation in primary microglia (Figure 4J–M).

Building upon these in vitro observations, we proceeded to recapitulate our key results using an MPTP‐induced mouse PD model in vivo. Mice were administered S3I‐201 (10 mg/kg/d) [18] by intraperitoneal (i.p.) injection and divided into four groups: (1) Vehicle + saline, (2) Vehicle + MPTP, (3) S3I‐201 + saline, and (4) S3I‐201 + MPTP. S3I‐201 significantly decreased TAB2 protein levels and suppressed p‐p65 activation in both the substantia nigra (Figure 4N, O) and striatum (Figure 4P, Q), although the suppression of p‐IκB was less pronounced. Notably, S3I‐201 significantly inhibited the increase of p‐STAT3 induced by MPTP in the substantia nigra but not in the striatum, revealing regional heterogeneity in pathway modulation and drug response. Importantly, despite the downregulation of TAB2 and partial attenuation of NF‐κB signaling, S3I‐201 did not prevent MPTP‐induced dopaminergic neuronal damage, as TH levels remained comparable between the MPTP and MPTP + S3I‐201 groups in both brain regions. These findings revealed the complexity underlying direct STAT3 inhibition. While systemic STAT3 downregulation modulated TAB2 expression and inflammatory pathways, this treatment was inadequate to elicit meaningful neuroprotective effects in the PD model. As TAB2 was conservatively upregulated in PD and its transcription was controlled by STAT3, as shown in our study, we hypothesized that specific brain TAB2 inhibition might alleviate inflammation and exert therapeutic effects in PD by protecting TH^+^ dopaminergic neurons.

### 2.5 *Tab2* Knockdown Exerted Neuroprotective Effects by Inhibiting the NF‐κB Pathway and Improving Behavioral Dysfunction in MPTP‐Induced PD mice

**FIGURE 5:**
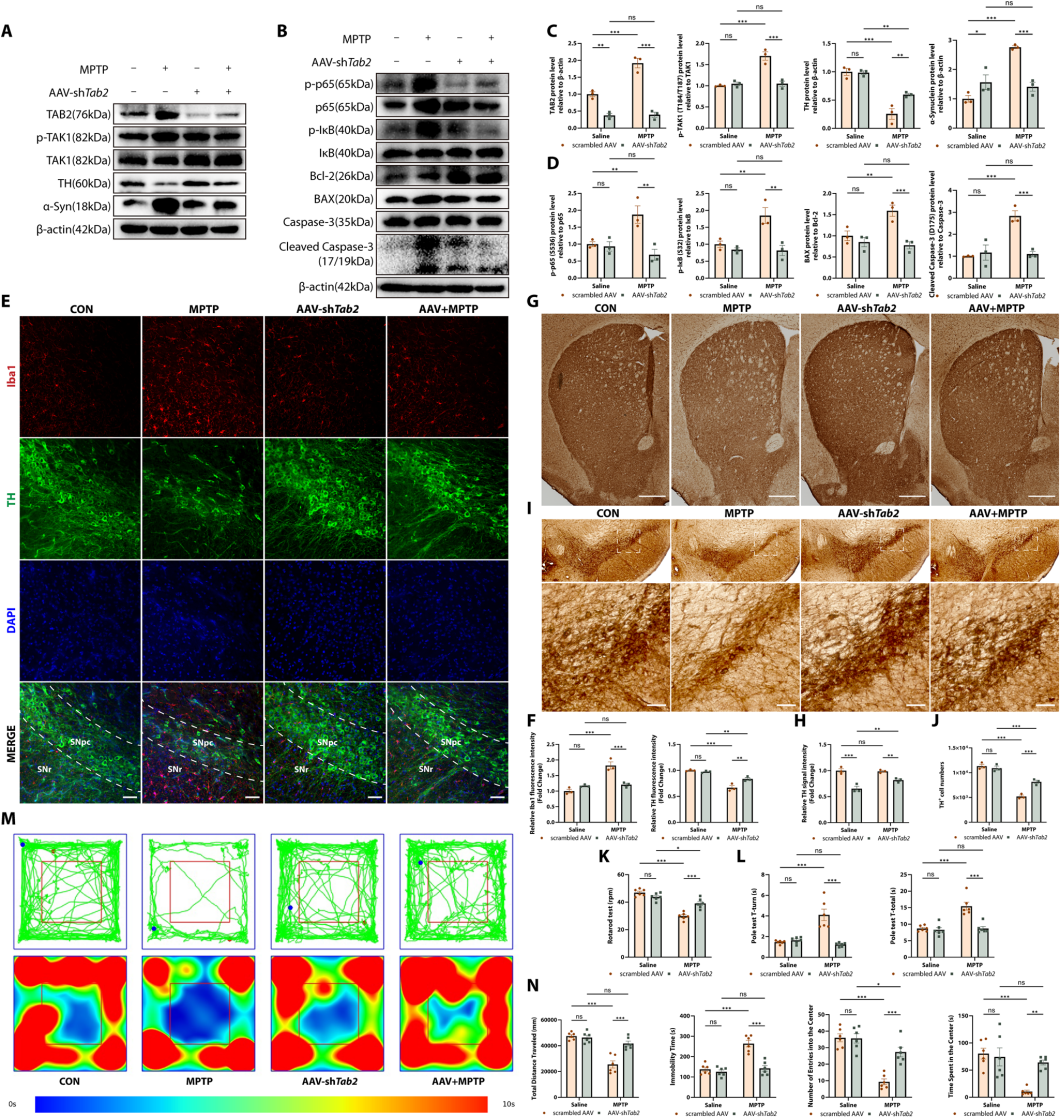
*Tab2* knockdown targeting microglia exerted neuroprotective effects and improved motor function in MPTP‐induced PD mouse models. (A–D) Expression of TAB2‐related (TAB2, p‐TAK1, TAK1), PD‐related (TH, α‐Syn), NF‐κB pathway (p‐p65, p65, p‐IκB, and IκB), and apoptosis pathway (Bcl‐2, BAX, Caspase‐3, cleaved Caspase‐3) proteins in the substantia nigra of mice receiving stereotaxic injection of microglia‐specific AAV‐control or AAV‐sh*Tab2* into the SNr, followed by subacute MPTP administration (*n* = 3). (E, F) Microglia and dopaminergic neurons were assessed by double staining of Iba1 and TH in the substantia nigra of mice (*n* = 3) (Scale bar, 50 µm). (G–J) TH^+^ dopaminergic fiber density in the striatum and neurons in the substantia nigra of mice (*n* = 3) (Scale bar, 500 µm, for striatum; Scale bar, 50 µm, for substantia nigra). (K–N) Motor function assessment in mice (*n* = 6). (K) Rotarod test (latency to fall, rpm). (L) Pole test: time to turn and total time to descend. (M) Representative movement trajectories in the OFT. (N) OFT parameters: total distance, immobility time, center entries, and time spent in the center. Quantitative data are presented as the mean ± SEM. ^*^ *p* < 0.05, ^**^ *p* < 0.01, and ^***^ *p* < 0.001.

To clarify the role of TAB2 expression in the pathogenesis of PD, we evaluated whether TAB2 alteration changed Parkinsonism in mice. Three AAV11‐*Iba1*p‐*EGFP*‐sh*Tab2* constructs were stereotaxically injected into the substantia nigra pars reticulata (SNr) of mice, and Western blot validation identified AAV‐sh*Tab2*‐1 as the most effective for suppressing TAB2 expression (Figure S5B,C). Immunofluorescence colocalization analysis revealed a Pearson correlation coefficient of 0.59 between Iba1 and EGFP, with approximately 75% of Iba1^+^ cells being EGFP^+^, indicating a transfection efficiency of approximately 75% (Figure S5D–F). Mice were divided into four groups: (1) scrambled AAV + saline, (2) scrambled AAV + MPTP, (3) AAV‐sh*Tab2* + saline, and (4) AAV‐sh*Tab2* + MPTP (Figure S5A). We found that both the number of TH^+^ neurons and TH protein levels were decreased in the substantia nigra of MPTP‐injected mice, when compared with saline‐treated controls, confirming that the model was successfully established. Western blot analysis revealed that MPTP injection significantly elevated TAB2 and p‐TAK1 levels and increased α‐Syn protein levels. We also found that the NF‐κB and apoptotic pathway components were activated in the MPTP‐treated mice. Nevertheless, *Tab2* knockdown significantly alleviated the MPTP‐induced decrease in TH levels and increase in α‐Syn protein levels, and suppressed both the NF‐κB and apoptotic pathways in the substantia nigra (Figure 5A–D).

To evaluate whether AAV‐sh*Tab2* targets microglia, co‐immunofluorescence staining of TAB2 with TH (marker of dopaminergic neurons) and Iba1 (specific marker of microglia) was performed. Results confirmed that TAB2 signal was indeed reduced in microglia (Iba1^+^) in the AAV‐sh*Tab2* group, whereas TAB2 expression in dopaminergic neurons (TH^+^) was not directly affected (Figure S5G,H). MPTP injection significantly increased Iba1 fluorescence intensity and decreased TH fluorescence intensity, indicating microglia activation and TH^+^ neuron injury. However, *Tab2* knockdown significantly inhibited microglia activation and protected TH^+^ neurons (Figure 5E, F). In addition, DAB staining showed that TH^+^ fiber density in the striatum was reduced in the MPTP group, and was significantly reversed by *Tab2* knockdown (Figure 5G, H). Quantification of TH^+^ neurons in the substantia nigra demonstrated a significant loss in the MPTP group, which was rescued by *Tab2* knockdown (Figure 5I, J).

To assess Parkinsonism‐related behavior, we used the rotarod test and pole tests, which are used to evaluate coordination and muscle strength. The falling rpm was reduced for MPTP‐treated mice, indicating motor impairment, which was reversed by *Tab2* knockdown (Figure 5K). In the pole test, the latency to turn and climb down the pole was prolonged in MPTP‐treated mice, and this was shortened by *Tab2* knockdown (Figure 5L). In the open field test (OFT), MPTP mice displayed motor deficits and anxiety‐like behaviors, as evidenced by decreased total distance, increased immobility time, and reduced center entries and duration. These behavioral abnormalities were reversed by *Tab2* knockdown (Figure 5M,N). Collectively, these data suggested that *Tab2* knockdown suppressed microglia activation, protected TH^+^ neurons, and improved motor dysfunction by inhibiting NF‐κB and apoptotic pathways.

### 2.6 *Tab2* Knockdown Reduced the Interaction Between TAB2 and α‐Syn, and Its Protein Expression was Suppressed by Lumacaftor

**FIGURE 6:**
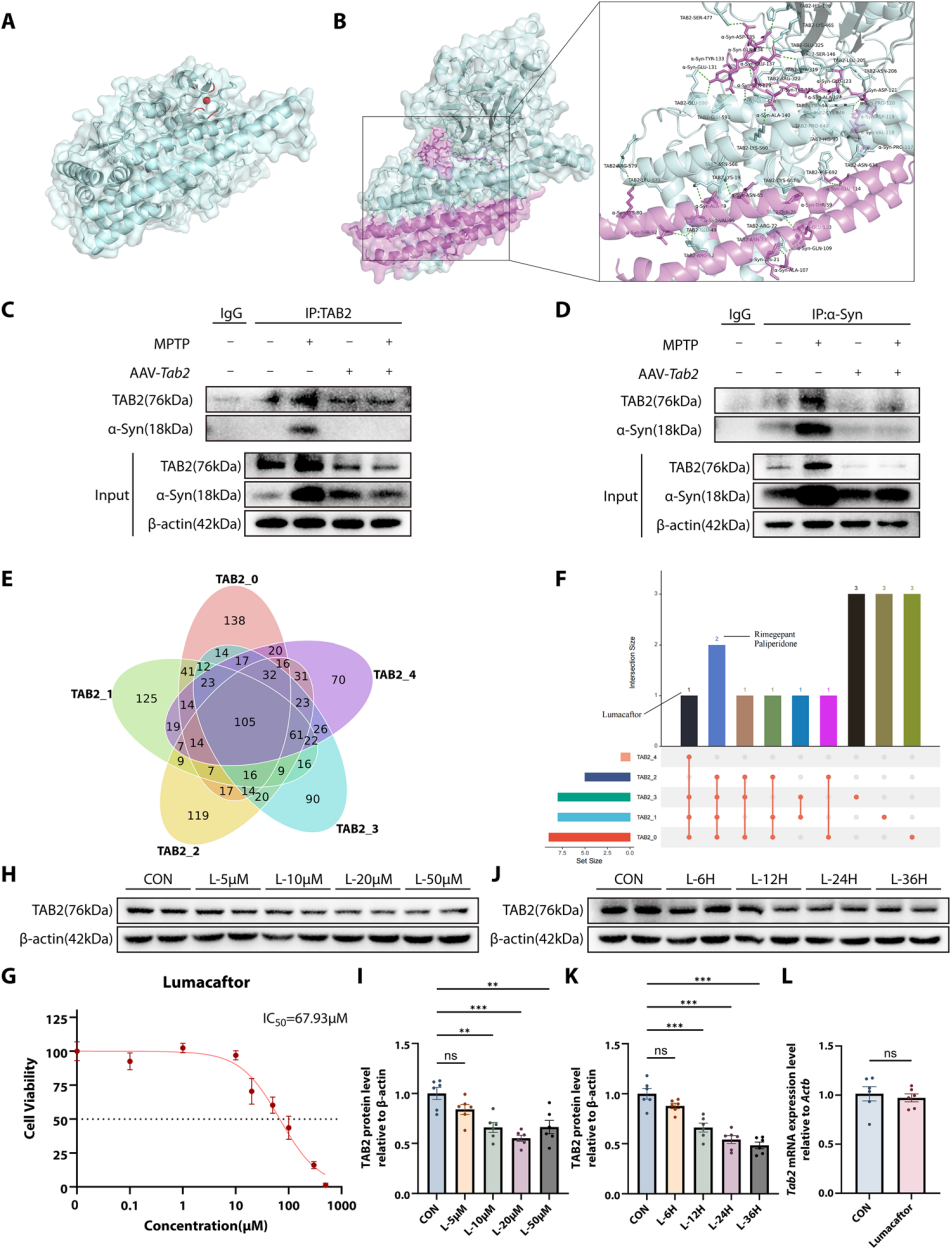
TAB2 interacted with α‐Syn, and its protein expression was suppressed by lumacaftor in BV‐2 cells. (A) Predicted structure of TAB2 generated using Protenix. The zinc finger motif within the NZF domain is highlighted in red, with red spheres indicating zinc ion sites and red lines marking the C4‐type zinc finger cysteine residues (Cys670, Cys673, Cys684, and Cys687) (pTM = 0.78). (B) Molecular docking using Protenix revealed the possible interaction sites of TAB2 and α‐Syn. Hydrogen bonds between interacting residues are shown as green dashed lines. (pTM = 0.88, ipTM = 0.91, ΔiG = −38.9 kcal/mol). (C, D) The interaction of TAB2 and α‐Syn in the substantia nigra was detected by co‐immunoprecipitation (Co‐IP). Tissue lysates were immunoprecipitated with anti‐TAB2 antibody (anti‐α‐Syn antibody) and probed with anti‐α‐Syn antibody (anti‐TAB2 antibody). (E) Intersection of MTiOpenScreen virtual screening results across Protenix‐predicted five TAB2 models. (F) DrugRep docking of five Protenix TAB2 models intersected with MTiOpenScreen predictions. Frequency scoring identified the three highest‐scoring drugs. (G) CCK‐8 assay results showing the dose‐dependent effect of lumacaftor on BV‐2 cell viability. The IC_50_ value was determined to be 67.93 µm (*n* = 6). (H, I) Dose‐dependent TAB2 expression in BV‐2 cells treated with lumacaftor for 36 h (*n* = 6). (J, K) Time‐dependent TAB2 expression in BV‐2 cells treated with lumacaftor at 10 µm (*n* = 6). (L) The mRNA expression of *Tab2* in BV‐2 cells treated with DMSO or lumacaftor was measured by RT‐qPCR (*n* = 6). Quantitative data are presented as the mean ± SEM. ^*^ *p* < 0.05, ^**^ *p* < 0.01, and ^***^ *p* < 0.001.

We first predicted the structure of TAB2 using Protenix (v0.4.6) (pTM = 0.78) (Figure 6A) [19]. Protein docking using Protenix revealed that TAB2 interacted with α‐Syn, a pathological hallmark of PD (pTM = 0.88, ipTM = 0.91, ΔiG = ‐38.9 kcal/mol) (Figure 6B). We therefore evaluated the effect of *Tab2* knockdown on the interaction between TAB2 and α‐Syn. Tissue extracts from the substantia nigra were immunoprecipitated with an anti‐TAB2 antibody or an anti‐α‐Syn antibody to detect the interaction between TAB2 and α‐Syn induced by MPTP. The results showed that *Tab2* knockdown reduced this interaction, which could be due to the reduction in total TAB2 protein rather than a specific disruption of the interaction (Figure 6C,D). However, these results did not rule out the occurrence of TAB2–α‐Syn binding nor diminish its potential contributions to the molecular underpinnings of PD pathology.

Next, we identified potential inhibitors of TAB2 to verify whether TAB2 could be an intervention target for the treatment of PD. The five TAB2 structural models predicted by Protenix were submitted to MTiOpenScreen for virtual screening against the Drug‐lib database [20]. Intersecting the top 500 drugs from each model identified 105 approved drugs (Figure 6E). We then submitted the five TAB2 models to DrugRep's Approved Drug Library for conventional AutoDock Vina docking analyses [21]. Intersecting these results with the 105 drugs from MTiOpenScreen revealed three compounds: lumacaftor, rimegepant, and paliperidone, which were present in four of the five lists (Figure 6F). These three drugs were then selected for further testing.

We performed CCK‐8 assays to determine the IC_50_ values of the three drugs in BV‐2 cells (Figure 6G; Figure S6A,B). To avoid cytotoxicity, sub‐IC_50_ concentrations were used to assess dose‐dependent effects on TAB2 expression. Lumacaftor significantly suppressed TAB2 protein expression at 10 µm, whereas rimegepant and paliperidone showed no effect on TAB2 levels (Figure 6H, I; Figure S6C–G). Time‐course analysis at 10 µm revealed that lumacaftor significantly inhibited TAB2 protein expression as early as 12 h post‐treatment (Figure 6J, K). RT‐qPCR analysis further revealed that lumacaftor treatment did not alter *Tab2* mRNA levels, indicating that this inhibitory effect was not mediated at the transcriptional level (Figure 6L).

### 2.7 Lumacaftor Inhibited TAB2‐Mediated NF‐κB Pathway Activation and Exerted Neuroprotective Effects in PD Cell Models

**FIGURE 7:**
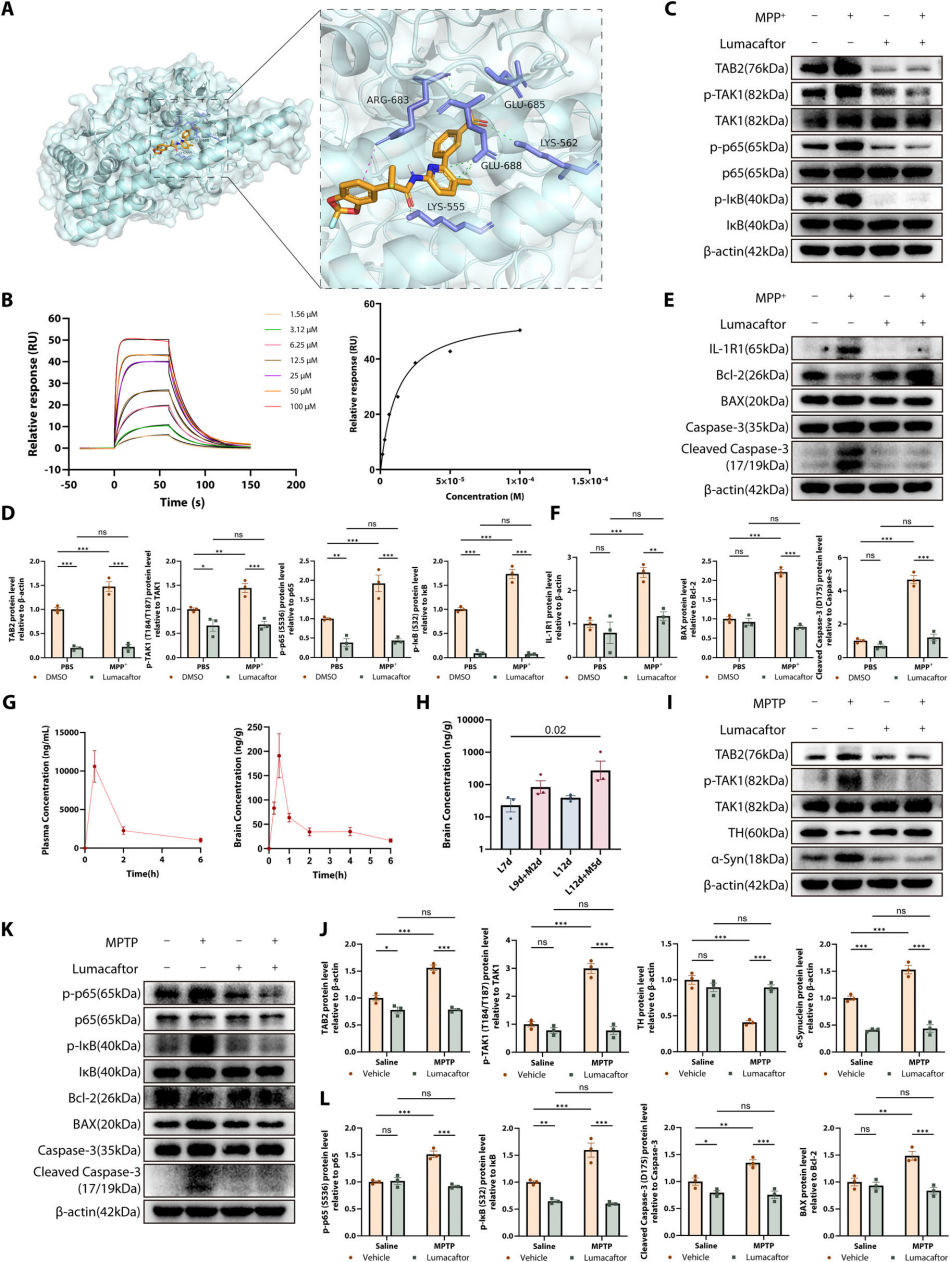
Lumacaftor inhibited TAB2‐mediated NF‐κB pathway activation and exerted neuroprotective effects in PD cells and mouse models. (A) Molecular docking simulation of lumacaftor bound to the NZF domain of TAB2. Green dashed lines denote hydrogen bond interactions, and pink dashed lines indicate *π–π* interactions between lumacaftor and key residues (Vina score = −7.9 kcal/mol). (B) SPR analysis showing the binding affinity of lumacaftor and the TAB2‐NZF domain. (C, D) Expression of TAB2‐related (TAB2, p‐TAK1, and TAK1), and NF‐κB pathway (p‐p65, p65, p‐IκB, and IκB) proteins in BV‐2 cells treated with DMSO [0.1% (v/v)] or lumacaftor (10 µm), followed by treatment with or without MPP^+^ (0.5 mm, 36 h) (*n* = 3). (E, F) Expression of IL‐1R1 and apoptosis pathway (Bcl‐2, BAX, Caspase‐3, and cleaved Caspase‐3) proteins in SH‐SY5Y cells co‐cultured with BV‐2 cells in a Transwell system. BV‐2 cells (upper chamber) were treated with DMSO [0.1% (v/v)] or lumacaftor (10 µm), followed by treatment with or without MPP^+^ (0.5 mm, 36 h) (*n* = 3). (G, H) Time‐dependent pharmacokinetic characteristics of lumacaftor in plasma and brain, as well as intergroup differences in brain drug accumulation following continuous administration between lumacaftor‐treated and lumacaftor + MPTP‐lesioned mice (*n* = 3). (I–L) Expression of TAB2‐related (TAB2, p‐TAK1, TAK1), PD‐related (TH, α‐Syn), NF‐κB pathway (p‐p65, p65, p‐IκB, and IκB), and apoptosis pathway (Bcl‐2, BAX, Caspase‐3, cleaved Caspase‐3) proteins in the substantia nigra of MPTP mouse models followed by lumacaftor pretreatment (*n* = 3). Quantitative data are presented as the mean ± SEM. ^*^ *p* < 0.05, ^**^ *p* < 0.01, and ^***^ *p* < 0.001.

Molecular docking revealed that lumacaftor bound to the NZF domain of TAB2, specifically at residues Lys555, Lys562, Arg683, Glu685, and Glu688 (Figure 7A). The C4‐type zinc finger motif of the TAB2‐NZF domain comprises Cys670, Cys673, Cys684, and Cys687. Binding to Arg683 and Glu685 could perturb the spatial positioning of Cys684, thereby disrupting the zinc finger structure and interfering with TAB2‐mediated signal transduction to block NF‐κB signaling.

To validate the predictions of molecular docking, we purified the TAB2‐NZF domain and performed surface plasmon resonance (SPR) experiments with lumacaftor. SPR analysis yielded an equilibrium dissociation constant (KD) of 1.13 × 10^−^^5^ m, with an association rate constant (ka) of 4.26 × 10^3^ m ^−^^1^s^−^^1^ and a dissociation rate constant (kd) of 4.80 × 10^−^^2^ s^−^^1^ (quality kinetics Chi^2^ = 0.55 RU^2^), indicative of moderate binding affinity. These results suggested that lumacaftor likely perturbed the ubiquitin‐recognition function of TAB2 by directly binding its NZF domain (Figure 7B).

We therefore observed the effect of lumacaftor on the NF‐κB signaling pathway. As indicated in prior experiments, cells were divided into four groups: (1) DMSO + PBS, (2) DMSO + MPP^+^, (3) lumacaftor + PBS, and (4) lumacaftor + MPP^+^. Western blot analysis showed that MPP^+^ activated the NF‐κB pathway when compared with the control group, whereas lumacaftor significantly attenuated NF‐κB pathway activation induced by MPP^+^ (Figure 7C, D).

To further evaluate whether lumacaftor rescued neuronal injury raised by MPP^+^‐induced neuroinflammation, BV‐2 and SH‐SY5Y cells were co‐cultured using Transwell inserts. Western blot analysis of SH‐SY5Y cells showed that lumacaftor significantly reduced IL‐1R1 protein levels and prevented the MPP^+^‐induced elevation of apoptosis pathway markers (Figure 7E, F). These results suggested that lumacaftor inhibited the TAB2‐mediated NF‐κB signaling pathway and protected neurons against injury.

### 2.8 Lumacaftor Suppressed the TAB2‐Mediated Neuroinflammatory Response by Disrupting its Recognition of K63‐Linked Ubiquitin Chains, and it Played a Neuroprotective Role and Improved Motor Function in MPTP‐Induced PD Mouse Models

Lumacaftor is an FDA‐approved drug used for treating cystic fibrosis. It was reported that lumacaftor treatment (3 mg/kg, i.p. injection) could rescue neuronal injury [22, 23, 24, 25]. We first evaluated the role of lumacaftor in TAB2 protein expression with a concentration gradient (1, 3, 5, and 10 mg/kg) and found that TAB2 suppression was already detectable in both the substantia nigra and striatum at 3 mg/kg (Figure S7). However, whether lumacaftor crosses the blood‐brain barrier (BBB) remains unclear. Therefore, a pharmacokinetic study was conducted. Lumacaftor reached its peak levels in plasma and brain tissue at 0.5 h after 3 mg/kg administration. The peak plasma and brain concentrations were 10,607 ng/mL and 191 ng/g, respectively, accompanied by rapid drug clearance (Figure 7G). These findings suggest that lumacaftor exhibits relatively low BBB permeability under physiological conditions. Notably, BBB integrity was destroyed in the MPTP‐induced PD model. Accordingly, lumacaftor was delivered via i.p. injection for 7 consecutive days prior to MPTP‐induced neurotoxic lesion establishment. In the lumacaftor‐treated groups, brain lumacaftor concentrations trended upward with prolonged administration, with mean brain tissue concentrations reaching 27.7 ng/g after 7 days of continuous injection and 39.9 ng/g after 12 days. Notably, brain lumacaftor accumulation was markedly augmented in the MPTP‐treated groups. The brain lumacaftor concentration increased to 104 ng/g at day 2 post‐MPTP exposure, which was higher than that detected in the 12‐day lumacaftor‐treated groups. The accumulation effect was further amplified at day 5 post‐MPTP administration, with the brain lumacaftor level peaking at 438 ng/g, equivalent to an approximately 11‐fold elevation relative to the 12‐day lumacaftor‐treated groups (Figure 7H). Collectively, these results demonstrate that BBB permeability of lumacaftor is significantly enhanced under the MPTP‐lesioned pathological states, which is expected to have a therapeutic effect. Thus, mice were divided into four groups: (1) Vehicle + saline, (2) Vehicle + MPTP, (3) lumacaftor + saline, and (4) lumacaftor + MPTP (Figure S8A), for subsequent behavioral and functional validation.

**FIGURE 8:**
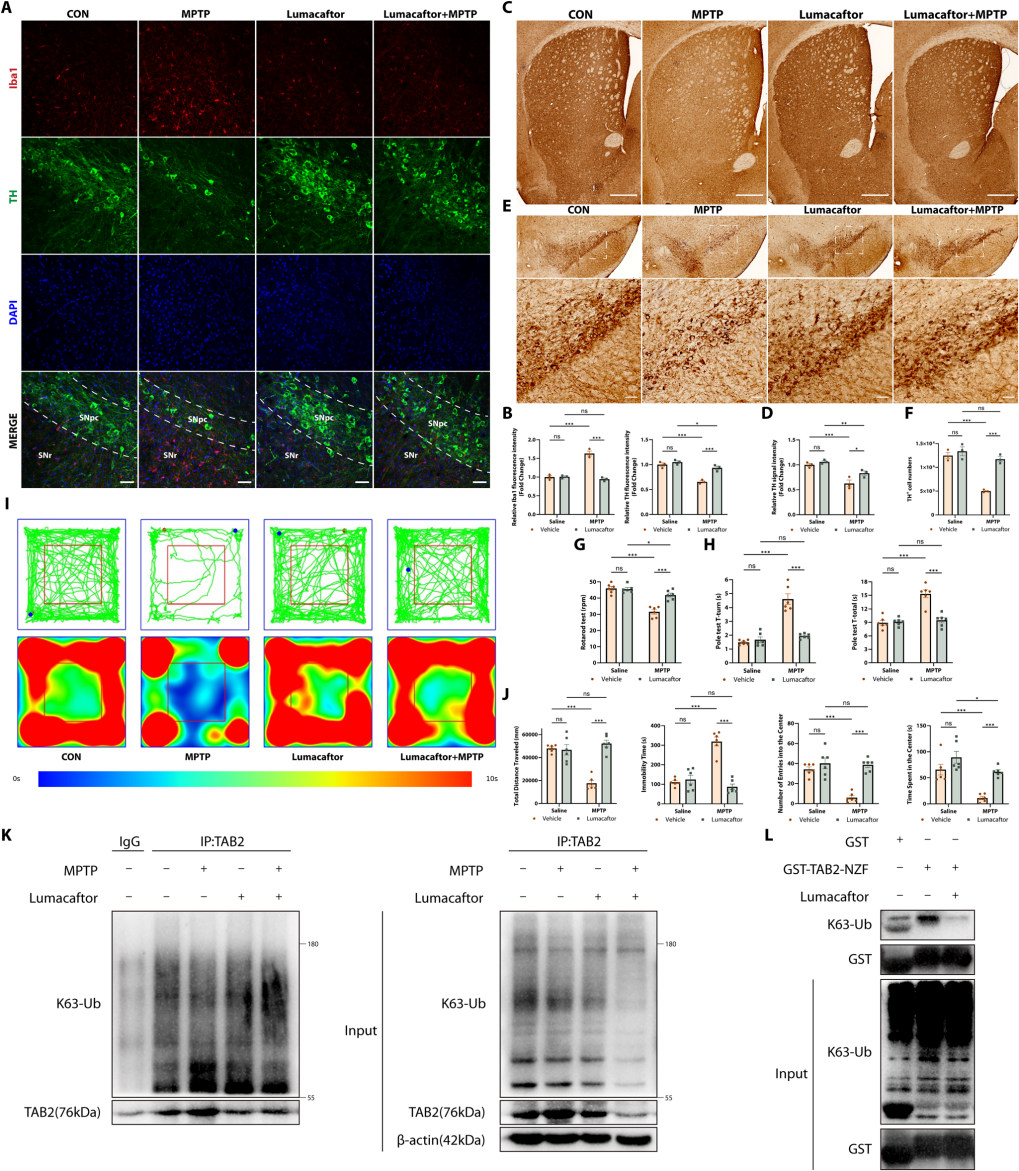
Lumacaftor suppressed TAB2 recognition of K63 ubiquitin chains, exerted neuroprotective effects, and improved motor function in MPTP‐induced PD mouse models. (A, B) Expression of Iba1^+^ microglia and TH^+^ dopaminergic neurons in the substantia nigra of MPTP mouse models followed by lumacaftor pretreatment (*n* = 3) (Scale bar, 50 µm). (C–F) TH^+^ dopaminergic fiber density in the striatum and TH^+^ dopaminergic neurons in the substantia nigra of mice (*n* = 3) (Scale bar, 500 µm, for striatum; Scale bar, 50 µm, for substantia nigra). (G–J) Motor function assessment in mice (*n* = 6). (G) Rotarod test (latency to fall, rpm). (H) Pole test: time to turn and total time to descend. (I) Representative movement trajectories in the OFT. (J) OFT parameters: total distance, immobility time, center entries, and time spent in the center. (K) Co‐IP analysis of K63‐linked ubiquitination of TAB2 in the substantia nigra. (L) GST pull‐down of K63‐linked ubiquitin chains by the GST‐tagged TAB2‐NZF protein. Quantitative data are presented as the mean ± SEM. ^*^ *p* < 0.05, ^**^ *p* < 0.01, and ^***^ *p* < 0.001.

Western blot analysis showed that lumacaftor injection significantly suppressed TAB2 and p‐TAK1 protein expressions, increased TH levels, decreased α‐Syn levels, and inhibited both NF‐κB and apoptotic pathway components in both the substantia nigra and striatum (Figure 7I–L; Figure S8B–E). Lumacaftor injection significantly prevented MPTP‐induced elevation of Iba1 fluorescence intensity and reduction of TH fluorescence intensity (Figure 8A, B). Moreover, immunofluorescence analysis showed that TAB2 signal was predominantly reduced in microglia (Iba1^+^) in the lumacaftor group, whereas TAB2 level in dopaminergic neurons (TH^+^) showed a relatively smaller change (Figure S8F,G). DAB staining revealed that lumacaftor significantly prevented MPTP‐induced reduction of TH^+^ fiber density in the striatum (Figure 8C, D) and MPTP‐induced loss of TH^+^ neurons in the substantia nigra (Figure 8E, F). Together, these results indicated that lumacaftor suppressed the TAB2‐mediated NF‐κB pathway and microglia activation, and protected TH^+^ neurons in MPTP‐induced PD mouse models.

We then assessed the impact of lumacaftor on Parkinsonism‐related behavior. Behavioral tests showed that lumacaftor injection significantly prevented MPTP‐induced motor dysfunction and anxiety‐like behaviors, specifically by increasing falling rpm on the rotarod, shortening turning time and total time in the pole test, and decreasing immobility time and increasing total distance, center entries, and time spent in the center in the OFT (Figure 8G–J).

As previously shown, lumacaftor bound to the NZF domain of TAB2, disrupting its zinc finger structure and thereby interfering with the recognition of K63 ubiquitin chains on upstream proteins such as TRAF6. We therefore performed TAB2 co‐immunoprecipitation (Co‐IP) and determined K63 ubiquitination levels in the associated proteins. K63 ubiquitination was significantly elevated in the MPTP‐injected group, while lumacaftor injection prevented this elevation, confirming our molecular docking predictions (Figure 8K). Subsequently, a recombinant GST‐tagged TAB2‐NZF domain was generated for GST pull‐down analysis. Our findings indicated that the TAB2‐NZF domain exhibited robust binding affinity for K63‐linked ubiquitin chains, and this binding interaction was significantly inhibited by lumacaftor in pull‐down eluates. Collectively, these results demonstrated that lumacaftor interfered with the recognition of K63 ubiquitin signals by the TAB2‐NZF domain (Figure 8L).

## 3 Discussion

The current study analyzed the anti‐inflammatory effect of *Tab2* knockdown/lumacaftor treatment against cell injury in PD cells and animal models. The results suggested that the suppressed microglia activation and inflammation by *Tab2* knockdown/lumacaftor treatment might correlate with the NF‐κB signaling pathway to promote the survival of dopaminergic neurons.

In the present study, we used sn‐RNA seq of the substantia nigra to depict the cellular landscape of PD, and identified microglia as the main disease‐associated cell population. Enrichment analysis showed that PD microglia were significantly enriched in inflammation‐related pathways, including the JAK‐STAT signaling pathway, Toll‐like receptor signaling pathway, TNF signaling pathway, NF‐κB signaling pathway, and NOD‐like receptor signaling pathway. Neuroinflammation is a well‐established feature of PD, and the role of microglia as central drivers of this process is increasingly recognized [26]. In PD, microglia exhibit a dual role; they are capable of clearing pathological α‐Syn aggregates but can also mediate neuroinflammatory injury to neurons [27]. Further microglia subclustering analysis revealed that the PD‐associated increase in microglia was not confined to a single subset, but was distributed across multiple microglial subpopulations, highlighting heterogeneous and potentially context‐dependent functions. This aligns with our evolving understanding of microglial biology. As Ransohoff stated in 2016, the simplistic M1/M2 dichotomy, borrowed from macrophage research, is inadequate for classifying microglia [28]. This view was formally consolidated in a 2022 consensus, which advocated for the dynamic “state” paradigm over the static “polarization” framework [29]. Our snRNA‐seq data supported this paradigm by demonstrating that microglia in PD did not uniformly adopt a monolithic “M1‐like” pro‐inflammatory state, but rather transitioned into a spectrum of distinct reactive states, emphasizing the complexity of their involvement in PD‐associated neuroinflammation and neuronal vulnerability. By integrating analyses, we prioritized *TAB2* as a key PD‐related microglial gene.

We found elevated *TAB2* transcript levels in midbrain microglia from single‐nucleus transcriptome data of PD patients, with conserved TAB2 upregulation across multiple PD animal and cellular models. To our knowledge, this is the first report of dysregulated *TAB2* transcription in midbrain microglia of PD patients. TAB2 facilitates the assembly of the TAK1–TABs complex, by recognizing K63‐linked ubiquitin chains, leading to activation of the NF‐κB pathway, which has been extensively validated across diverse immune responses [30, 31]. Single‐nucleus analysis revealed that TAB2 elevation occurred in both MC1 and MC2 microglia from PD patients. Monocle3 trajectory analysis further uncovered a continuous transition from resting MC1 microglia to the pro‐inflammatory MC2 state, in which TAB2 expression remained elevated, supporting the role of TAB2 as an initiating inflammatory driver of pathological processes underlying PD. This aligned with our observation that TAB2 expression peaked at 36 h before declining, while inflammatory pathways remained persistently activated, indicating an initiating role of TAB2 in neuroinflammation, as well as establishing its potential as an early‐stage biomarker for PD. Importantly, while the NF‐κB pathway was significantly activated by MPP^+^ stimulation, the MAPK pathway was concurrently inhibited, which was a phenomenon contrary to expectations, but had been reported by others [32], emphasizing the complexity of inflammatory pathways and MPP^+^‐driven NF‐κB‐specific inflammation.

Our findings showed that *Tab2* knockdown caused a fundamental shift in microglial polarization toward an anti‐inflammatory phenotype, which remained stable even during MPP^+^ challenge, thereby preventing the transition to a pro‐inflammatory state that typically released neurotoxic cytokines and induced neuronal damage. Dysregulated microglial activation perpetuates a vicious cycle of neurodegeneration through sustained release of proteases, pro‐inflammatory cytokines, and reactive oxygen species, ultimately leading to dopaminergic neuron loss [33]. In our experiments, *Tab2* knockdown in microglia exerted anti‐inflammatory and neuroprotective effects, with IL‐1β identified as an inflammatory mediator regulated by TAB2. Multiplex cytokine profiling using a Luminex assay revealed consistent upregulation of five key cytokines (IFN‐γ, IL‐12p70, IL‐1β, IL‐6, and GM‐CSF) following MPP^+^ treatment in both intracellular compartments and culture media, which was attenuated upon *Tab2* knockdown. In contrast, IL‐6 and TNF‐α did not show corresponding transcriptional changes during qPCR analysis, a discrepancy likely attributable to the higher sensitivity of the Luminex assay in detecting low‐abundance secreted proteins, and to post‐transcriptional regulatory mechanisms that may have decoupled mRNA expression from protein secretion, given the 36 h treatment duration. Using a Luminex assay, we measured 12 cytokines (IFN‐γ, IL‐12p70, IL‐13, IL‐1β, IL‐2, IL‐4, IL‐5, IL‐6, TNF‐α, GM‐CSF, IL‐18, and IL‐10) in both cell lysates and culture media. Only five cytokines showed consistent changes in both compartments, while others displayed either high intracellular levels but low secretion or no significant changes (data not shown), highlighting the complexity of MPP^+^‐induced inflammation and TAB2 regulation. Importantly, IL‐1β exhibited consistent and robust modulation in both qPCR and Luminex assays, emphasizing its important role as a downstream effector of TAB2 signaling. Collectively, these results indicated that microglial *Tab2* knockdown resulted in neuroprotection by disrupting the microglial inflammatory cascade, with IL‐1β identified as an inflammatory factor regulated by TAB2.

STAT3 elevation has been consistently demonstrated in multiple PD studies [34, 35]. Although extensively investigated in oncology using numerous inhibitors, STAT3‐targeted therapies have largely failed clinical trials, primarily due to the broad gene regulation of STAT3 as a transcription factor for many proteins [36]. Similarly, direct STAT3 inhibition acted as a double‐edged sword in PD, as it alleviated microglial neuroinflammation but exacerbated protein aggregation in astrocytes [37, 38]. Consistently, intraperitoneal STAT3 inhibition suppressed TAB2 and inflammation but failed to restore TH, unlike the robust neuroprotection of TAB2‐specific targeting. Despite the established microglial STAT3–TAB2 axis, divergent outcomes reveal that STAT3 exerts TAB2‐independent pleiotropic effects. Systemic STAT3 blockade disturbs multiple counteractive pathways. Accordingly, TAB2‐targeted intervention constitutes a more accurate therapeutic strategy for PD, avoiding adverse off‐target effects of pan‐STAT3 inhibition.

Using protein prediction and molecular docking, we first predicted TAB2 structure, and identified its interaction with the PD pathological hallmark, α‐Syn, which was confirmed by Co‐IP, further emphasizing the importance of TAB2 in the development of PD. The α‑Syn‑driven activation of microglia and astrocytes elicits a cascade of inflammatory signaling, particularly via IL‑1β and TNF‑α, which exacerbates neuronal vulnerability and accelerates disease progression [39]. Our Co‑IP results were consistent with these findings, and suggested that TAB2 may participate in α‑Syn‑mediated neuroinflammatory pathways, suggesting a functionally significant interaction between the two molecules during the progression of PD. We also identified lumacaftor as a TAB2 inhibitor using virtual screening, confirming its ability to suppress TAB2 expression, both in vitro and in vivo. Lumacaftor, an FDA‐approved cystic fibrosis drug, stabilizes mutated Cystic Fibrosis Transmembrane Conductance Regulator (CFTR) to prevent degradation [40]. Whether microglia in the substantia nigra of PD patients harbor CFTR structural alterations similar to those seen in cystic fibrosis remains unknown, but warrants further investigation. Lumacaftor is a first‐generation CFTR corrector that rescues the misfolded F508del‐CFTR protein, enabling its trafficking to the cell surface. Clinically, lumacaftor is used in combination with the potentiator, ivacaftor, for F508del‐homozygous patients. Lumacaftor‐ivacaftor therapy has demonstrated significant and sustained anti‐inflammatory effects in 44 F508del‐homozygous adults with cystic fibrosis, over 12 months. Treatment led to reductions in key proinflammatory cytokines in both the systemic circulation (plasma IL‐8, TNF‐α, and IL‐1β) and airway compartments (sputum IL‐6, IL‐8, IL‐1β, and TNF‐α), while anti‐inflammatory IL‐10 remained unchanged [41]. In the context of systemic disease‐induced neurodegeneration, myocardial infarction (MI) triggers neuroinflammation that downregulates CFTR in hippocampal neurons, causing synaptic loss and memory deficits. Lumacaftor, either alone or combined with ivacaftor, restores neuronal CFTR levels, normalizes hippocampal structure, and rescues memory dysfunction even when administered weeks after MI, emphasizing its therapeutic potential beyond cystic fibrosis [22]. However, the potential effects of lumacaftor in PD remain completely unexplored.

Although several studies have investigated the effects of lumacaftor in neurological diseases, none have evaluated its permeability across the BBB [22, 23, 24, 25]. As a highly plasma protein‐bound drug, lumacaftor was predicted to poorly permeate the BBB. Unexpectedly, our pharmacokinetic analyses demonstrated that lumacaftor can permeate the BBB at measurable concentrations under physiological conditions. Intriguingly, brain concentrations of lumacaftor were elevated ∼11‐fold in MPTP‐induced PD model mice, thereby supporting its promising therapeutic value for PD treatment.

Our molecular docking analysis predicted that lumacaftor directly bound to the NZF domain of TAB2. This structural prediction was functionally validated by our in vitro and in vivo experiments, which demonstrated that lumacaftor effectively inhibited TAB2 activity, suppressed microglial neuroinflammation, and consequently protected dopaminergic neurons from inflammatory damage. We propose that lumacaftor may similarly engage the NZF domain of TAB3, to prevent TAB2/3 from recognizing upstream K63‐linked ubiquitin chains. This could disrupt TAK1–TABs complex assembly and impair NF‐κB pathway activation. Collectively, these findings suggest the therapeutic potential of lumacaftor for the treatment of NF‐κB‐driven neuroinflammatory conditions. The NZF domain of TAB2 has been shown to recognize K63‐linked polyubiquitin chains on upstream adaptors, thereby recruiting TAK1 and activating NF‐κB signaling. Studies have shown that TAB2, as a sensor, detects K63 ubiquitin in neuroinflammatory cascades. Specifically, TRIM45 enhanced K63‐linked ubiquitination to activate NF‐κB [42], whereas USP25 attenuated the K63 ubiquitin to inactivate NF‐κB [14]. These opposing modulations emphasized the pivotal function of TAB2 in inflammatory signals. Although a recent study reported that TAB2 deficiency did not interfere with intact TAK1‐dependent NF‐κB and MAPK signaling, these findings were limited by the exclusive use of non‐immune cells and the absence of in vivo validation [43]. Such a conclusion contradicts the embryonic lethality observed in *Tab2* knockout mice and congenital heart disease caused by human TAB2 mutations, which are both attributed to impaired NF‐κB signaling [44, 45, 46]. Nevertheless, the discovery of a TAK1‐independent, cytoprotective function for TAB2 in TNF signaling emphasized the multifaceted nature of TAB2 beyond its canonical role in initiating TAK1‐mediated NF‐κB activation. This mechanistic complexity was observed in our neuroinflammatory model, where additional regulatory layers remain to be elucidated.

Neuroinflammation is one of the key contributors to PD pathogenesis, resulting in dopaminergic neurodegeneration and progression of the disease. As reported in several studies, evidence of the role of neuroinflammation in PD patients is indicative of higher levels of proinflammatory cytokines in serum and cerebrospinal fluid, and the presence of activated microglia in different CNS areas, such as the substantia nigra [47]. Our in vitro and in vivo results demonstrated that both genetic knockdown and pharmacological inhibition of TAB2 suppressed neuroinflammation, conferred robust neuroprotection, and significantly ameliorated motor dysfunction and anxiety‐like behaviors in PD models. These findings aligned with accumulating evidence that targeting neuroinflammation represents a viable therapeutic strategy for PD, highlighting its significant clinical potential [48].

**FIGURE 9:**
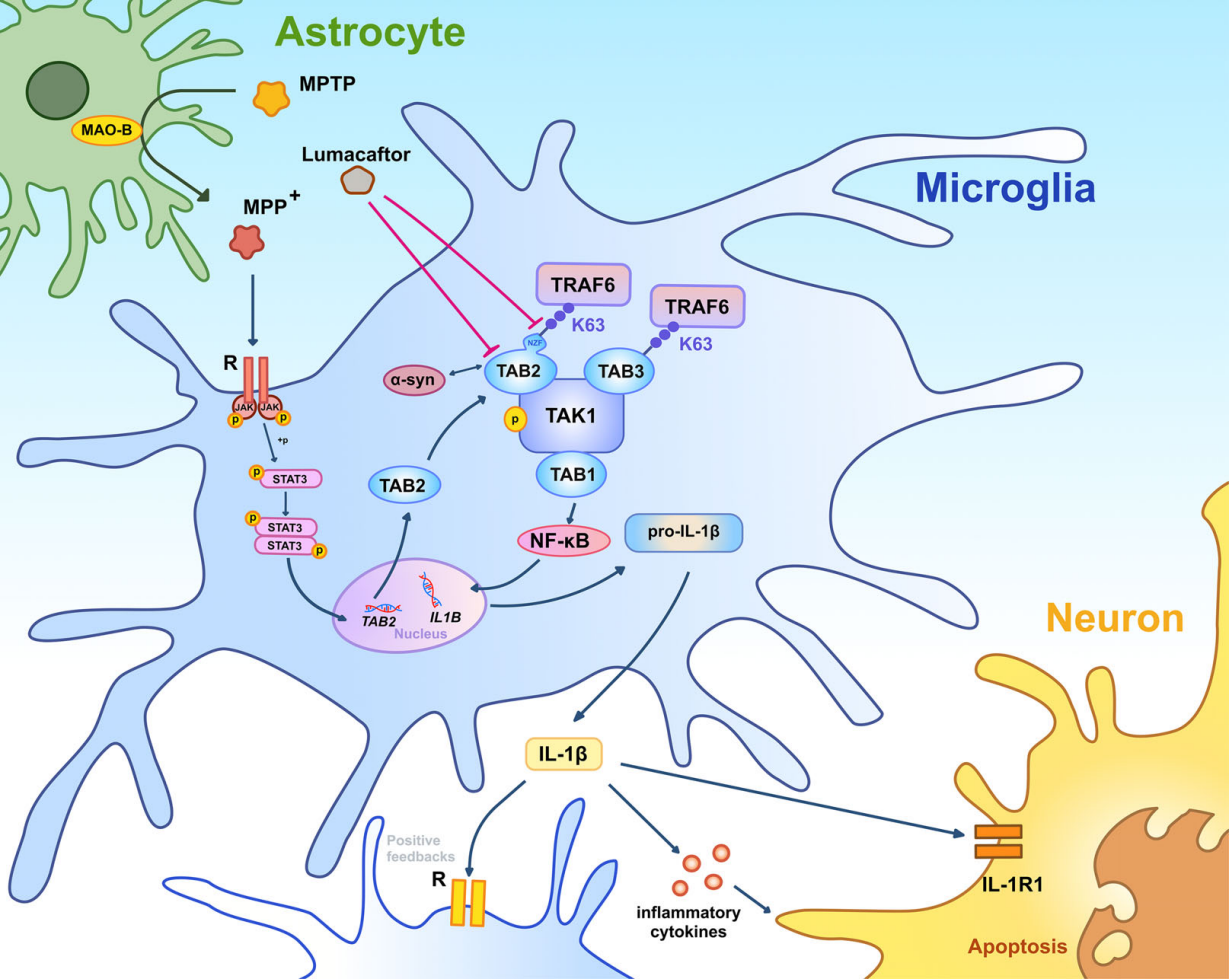
Schematic diagram of TAB2‐mediated neuroinflammation and the neuroprotective mechanism of lumacaftor against inflammation‐mediated neuronal injury in PD models. In microglia, STAT3 leads to the upregulation of TAB2 expression. TAB2 promoted NF‐κB pathway activation through its NZF domain‐mediated recognition of K63‐linked ubiquitin chains, leading to the release of inflammatory cytokines and subsequent neuronal injury. Lumacaftor exerted neuroprotective effects by suppressing TAB2 protein expression and directly binding to the TAB2‐NZF domain to interrupt K63 ubiquitin recognition, thereby blocking the NF‐κB signaling pathway and neuroinflammation.

In summary, this study showed that TAB2 caused neuronal damage by aggravating microglia‐mediated neuroinflammation during PD. However, downregulation of TAB2 (*Tab2* knockdown or use of the TAB2 inhibitor, lumacaftor) suppressed neuroinflammation and exerted neuroprotection in PD animal and cellular models, by interfering with its K63 ubiquitin chain recognition and inactivating the TAK1–TABs complex, resulting in the inhibition of the NF‐κB pathway and reduced release of inflammatory cytokines (Figure 9). These results provided insight into the role of TAB2 in the pathogenesis of PD, and potentially provided novel targets for pharmacological treatment strategies targeting TAB2.

## 4 Experimental Section

### 4.1 The snRNA‐Seq Data Processing

We downloaded the snRNA‐seq dataset GSE157783 from the Gene Expression Omnibus (GEO), including midbrain samples from five patients with PD and six HCs [49]. Following standard procedures, the data were processed using the Seurat software package [50]. As a quality control step, we removed cell nuclei from each sample that had fewer than 200 genes, a high nUMI threshold (range > 5000–7000), or a mitochondrial percentage > 10%. The top 2000 genes with the highest variability were used for PCA, and then the top 20 principal components were selected for subsequent analysis. Batch correction was performed using the Harmony algorithm to reduce non‐biological variation between samples. We then visualized the data using UMAP. Differential expression across cell types and subclusters was performed in Seurat using a negative binomial model. Genes were considered significant if they passed false discovery rate (FDR) correction (*p* < 0.05), and showed a log‐fold change > 0.25. The miloR was used to detect differential abundance between groups [51]. The hdWGCNA was performed to identify key gene co‐expression modules in PD microglial samples [52]. The “CellChat” package provided an effective framework for analyzing intercellular interactions and communication [53]. We used CellChat to infer cell‐cell signaling interactions among midbrain cell types, and to quantify the interaction probabilities and their statistical significance.

### 4.2 Bulk Transcriptome Data Analysis

This study used bulk transcriptome data downloaded from the GEO database, including GSE7621, GSE20141, GSE49036, GSE20186, GSE20295, and GSE8397. In total, the bulk transcriptome data comprised 81 PD substantia nigra samples and 65 HCs. Batch correction was performed using the combat function in the “sva” package [54]. Candidate genes identified from the snRNA‐seq analysis were then matched to the integrated bulk transcriptomic expression matrix. Genes that were absent from the bulk dataset, duplicated after gene‐symbol conversion, or failed to pass expression filtering were excluded. The remaining candidate genes were used as input variables for LASSO regression analysis to further reduce the number of candidate genes and minimize potential confounding effects [55].

### 4.3 Candidate Gene Screening

Candidate genes were identified by integrating differential expression analyses and hdWGCNA results. Differential expression analysis between PD and control samples was performed using the FindMarkers function in Seurat both across all cell populations and within the microglial subset. Genes with log_2_FC > 0.25 and adjusted *p*‐value < 0.05 were considered significantly differentially expressed. In parallel, hub genes were extracted from the PD‐associated hdWGCNA module, and the top 25 genes were selected. The overlap among the whole‐dataset DEGs, microglia‐specific DEGs, and hdWGCNA hub genes was determined using Venn analysis, yielding candidate genes for subsequent feature selection analyses.

### 4.4 Cell Culture and Transfection

BV‐2 and SH‐SY5Y cells were purchased from Pricella Biotechnology Co., Ltd (Wuhan, China). The cells were cultured in Dulbecco's Modified Eagle's Medium (DMEM) (C11995500BT; Thermo Fisher Scientific, Waltham, MA, USA) supplemented with 1% penicillin/streptomycin and 10% fetal bovine serum (FBS) (10091‐148; Thermo Fisher Scientific). Three siRNAs targeting *Tab2* (Table S1) (Guangzhou Ruibo Bio Co., Ltd., Guangzhou, China) were transfected into BV‐2 cells using Lipofectamine 3000 (L3000‐015; Thermo Fisher Scientific) in Opti‐MEM (31985‐070; Thermo Fisher Scientific) according to the instructions of the manufacturer. Cells were incubated at 37°C in a humidified atmosphere of 5% CO_2_ for the indicated times. The drugs used for cell treatments included LPS (L6529; Sigma–Aldrich, St. Louis, MO, USA), α‐Syn PFFs (SPR‐322; StressMarq Biosciences, Victoria, BC, Canada), S3I‐201 (S1155; Selleck Chemicals, Houston, TX, USA), and lumacaftor (S1565; Selleck Chemicals).

### 4.5 Cell Viability Assay

Cell viability was assessed using the Cell Counting Kit‐8 (B34304; Selleck Chemicals). Briefly, cells were seeded in 96‐well plates at a density of 2 × 10^3^ cells/well and allowed to adhere overnight. After treatment with different concentrations for 36 h, the culture medium was replaced with 100 µL fresh DMEM containing 10% CCK‐8 reagent. Following incubation at 37°C for 1 h, absorbance was measured at 450 nm using a microplate reader (Infinite M200 Pro, Tecan, Switzerland). Cell viability was expressed as a percentage of the control group after background subtraction.

### 4.6 Primary Microglial Culture

Primary cultured microglia were prepared from the cerebral cortices of C57BL/6 mice (postnatal days 0–1) by stripping the meninges and blood vessels. Brain tissues were minced into small fragments and triturated gently, followed by filtration through a 40 µm cell strainer to remove undigested tissue debris. Cell suspensions were centrifuged at 1000 rpm for 5 min at room temperature. The cell pellet was resuspended in complete medium [DMEM/F12 (C11330500BT; Thermo Fisher Scientific) supplemented with 1% penicillin/streptomycin and 10% FBS] and seeded into T75 cell culture flasks at a density of 1 × 10^6^ cells per flask. Cells were incubated in a humidified incubator with 5% CO_2_ at 37°C. Half of the culture medium was replaced every 3 days. After 14 days of mixed glial culture, confluent flasks were subjected to orbital shaking to isolate microglia. Culture flasks were fixed on a horizontal shaker and shaken at 180 rpm for 2 h at 37°C. Detached microglia‐rich supernatant was collected and centrifuged at 1000 rpm for 5 min. The cell pellet was resuspended in fresh complete medium and seeded onto cell culture plates or coverslips at the required density for subsequent experiments. Immunofluorescence staining for Iba1, a specific biomarker of microglia, was performed to verify cell purity. Cultures with an Iba1^+^ proportion exceeding 95% were used for follow‐up functional assays.

### 4.7 Animals and Ethics Approval

Male C57BL/6J mice and male Sprague–Dawley rats were obtained from Beijing HFK Bioscience Co., Ltd. (Beijing, China). Animals were housed under controlled conditions (22°C–24°C, 12 h light/dark cycle) with free access to food and water. All experimental procedures were approved by the Ethics Committee of Beijing Neurosurgical Institute (Process No. 202202014 and 202203024).

### 4.8 Construction and Stereotactic Injection of *Tab2* Knockdown Adeno‐Associated Virus (AAV)

Three shRNA sequences targeting the mouse *Tab2* gene were constructed into an RNAi vector using the *EGFP*‐mir155 skeleton driven by the *Iba1*p promoter, and the virus was packaged with the AAV11 serotype. Finally, AAV11‐*Iba1*p‐*EGFP*‐sh*Tab2* adeno‐associated virus, which specifically knocks down the *Tab2* gene in microglia, was obtained (Table S2) (1 × 10^13^ vg/mL, GeneChem, Shanghai, China). The vector carrying the *EGFP* reporter gene was used to help determine the infection efficiency. The AAVs, including scrambled AAV and AAV11‐*Iba1*p‐*EGFP*‐sh*Tab2*, were bilaterally microinjected into the SNr at the following coordinates: AP − 3.15 mm, ML ± 1.5 mm, DV − 4.5 mm. Mice were injected with 1.0 µL of AAV at a rate of 0.2 µL/min using a stereotaxic apparatus. Three weeks postinjection, mice were treated with MPTP to develop PD models. Animals were randomly assigned to experimental groups.

### 4.9 PD Model Establishment

The mouse PD model was induced by i.p. injection of MPTP hydrochloride (20 mg/kg free base) (S4732; Selleck Chemicals) dissolved in saline for 5 consecutive days. Control mice received equivalent volumes of saline. The rat PD model was established by stereotaxic injection of 6‐OHDA (H4381; Sigma–Aldrich) into the left striatum. The 6‐OHDA was prepared at 5 µg/µL in a solution containing 0.02% ascorbic acid and 0.9% saline, and administered at two sites: AP + 1.6 mm, ML + 2.4 mm, DV − 4.2 mm; and AP − 0.2 mm, ML + 2.6 mm, DV − 7.0 mm. A 2‐µL volume of 6‐OHDA or vehicle was injected at 0.5 µL/min at each site (total dose = 20 µg over two sites), with the vehicle injection serving as a control. After injection, the needle was left in place for 5 min before being slowly withdrawn. The skin was sutured and rats were allowed to recover from the surgery for 14 days. Contralateral rotations were measured following subcutaneous injection of apomorphine (0.5 mg/kg). Rats exhibiting > 60 contralateral rotations per 30 min were included as successful PD rat models.

To validate the regulation of TAB2 expression and the NF‐κB signaling pathway by STAT3 in vivo, mice were randomly divided into four groups: (1) Vehicle + saline, (2) Vehicle + MPTP, (3) S3I‐201 + saline, and (4) S3I‐201 + MPTP. Following 7 days of i.p. pretreatment with S3I‐201 (10 mg/kg), a subacute MPTP model was established by daily MPTP injection for 5 consecutive days, during which S3I‐201 (10 mg/kg) was continuously administered. Mice were sacrificed one week after the final MPTP injection.

To evaluate the role of TAB2 expression on the pathology of PD, mice were randomly assigned to (1) scrambled AAV + saline, (2) scrambled AAV + MPTP, (3) AAV‐sh*Tab2* + saline, and (4) AAV‐sh*Tab2* + MPTP. All groups received a stereotaxic injection of AAV into the SNr. At 3 weeks post‐surgery, mice were subjected to 5‐day i.p. MPTP injections, followed by behavioral testing and sacrifice. The experimental design is shown in Figure S5A.

To investigate the effect of lumacaftor on neuroprotection in PD, mice were randomly divided into four groups: (1) Vehicle + saline, (2) Vehicle + MPTP, (3) lumacaftor + saline, and (4) lumacaftor + MPTP. After 7 days of i.p. lumacaftor (3 mg/kg) pretreatment, a subacute MPTP model was performed by injecting MPTP for 5 consecutive days, also with lumacaftor (3 mg/kg) injection, followed by behavioral testing and sacrifice. The experimental design is shown in Figure S8A.

### 4.10 Behavioral Tests

All behavioral tests were preceded by 2–3 days of pretraining, followed by formal assessment. Investigators were blinded to treatment groups throughout all behavioral experiments.

### 4.11 Pole Test

Bradykinesia and motor performance were evaluated using a wooden pole (50 cm height, 1 cm diameter) with a 1.5‑cm ball at the top. During pretraining, mice were placed on the pole for three trials per day to familiarize them with the task. For formal testing, the time taken to turn and descend (t‑turn) and the total time required to reach the base of the pole (t‑total) were recorded for each mouse.

### 4.12 Rotarod Test

Motor coordination and balance were assessed using a five‐channel accelerating rotarod apparatus. Animals were pretrained for 3 consecutive days at a constant speed of 20 rpm for 5 min per day. During formal testing, mice were placed on the rotating drum with acceleration from 5 to 50 rpm within 5 min, with a maximum trial duration of 10 min. The total time and the rotational speed (rpm) at the time of falling were recorded automatically.

### 4.13 Open Field Test

Spontaneous locomotor activity was measured in an open field arena (50 cm × 50 cm × 40 cm). Mice were pretrained by allowing them to explore the arena for 10 min daily for 2 days. During the formal test, mice were placed in the center of the arena, and their spontaneous activities were recorded for 10 min using the VisuTrack system (Shanghai Xinruan Information Technology Co., Ltd., Shanghai, China).

### 4.14 Pharmacokinetic Analysis

For in vivo pharmacokinetic profiling, healthy and MPTP‐lesioned C57BL/6 mice were injected i.p. with 3 mg/kg lumacaftor. Plasma and brain tissue samples were harvested at sequential post‐administration time intervals. All biological samples were stored at −80°C until analysis to avoid compound degradation. After protein precipitation pretreatment, lumacaftor concentrations in biological matrices were quantified by Shimadzu high‐performance liquid chromatography coupled with tandem mass spectrometry. Concentration‐time curves were plotted based on calibrated standard curves.

### 4.15 Western Blot Analysis

Tissues or cells were washed with ice‐cold PBS and lysed in radioimmunoprecipitation assay (RIPA) buffer [50 mm Tris‐HCl (pH 7.4), 150 mm NaCl, 1% Nonidet P‐40, 0.1% SDS] supplemented with phosphatase and protease inhibitor cocktails (524628 and 539134; Sigma–Aldrich). Homogenates were centrifuged at 12 000 × *g* for 20 min at 4°C. Protein concentrations in supernatants were determined using a bicinchoninic acid (BCA) protein assay kit (23227; Thermo Fisher Scientific) according to the manufacturer's instructions. Equal amounts of protein (30 µg) were resolved by SDS‐PAGE on 8%–12% polyacrylamide gels and transferred to PVDF membranes (ISEQ00010; Millipore, Cork, Ireland). Membranes were blocked with 10% non‐fat milk for 1 h, then incubated overnight at 4°C with primary antibodies (Table S3). After washing, membranes were incubated with HRP‐conjugated secondary antibodies (ANM02‐1 and ANR02‐1; NeoBioscience, Shenzhen, China; 1:10 000) for 1 h. Protein bands were visualized using enhanced chemiluminescence substrate, and signal intensity was quantified using ImageJ software (National Institutes of Health, USA).

### 4.16 Co‐IP

Tissue lysates were prepared using immunoprecipitation (IP) lysis buffer (87787; Thermo Fisher Scientific) supplemented with protease inhibitor cocktail, phosphatase inhibitors, and NEM (E3876; Sigma–Aldrich). Protein concentration was determined using the BCA assay. For each IP, 500 µg of protein was incubated with primary antibody or control IgG overnight at 4°C. Subsequently, 20 µL of Protein A/G magnetic beads (88803; Thermo Fisher Scientific) were added and incubated for 1 h at RT. Beads were washed four times with ice‐cold wash buffer using magnetic separation (1 min per wash) to collect the beads. Immune complexes were eluted by boiling in SDS loading buffer for 10 min at 95°C. The supernatant was analyzed by Western blotting.

### 4.17 GST Pull‐Down Assay

The GST‐TAB2‐NZF fusion protein (amino acids 663–693 of mouse TAB2, UniProt: Q99K90) was expressed and purified. The purified protein was bound to glutathione magnetic beads (abs50082; Absin, Shanghai, China) and incubated with BV‐2 cell lysates supplemented with protease inhibitor cocktail and NEM, in the presence or absence of lumacaftor, overnight at 4°C. After magnetic separation and washing, bound proteins were eluted and analyzed by Western blotting.

### 4.18 Immunohistochemistry (IHC) Analysis

For IHC staining, the brain was removed and dehydrated in 20% and 30% sucrose solution, then cut into sections at a thickness of 40 µm, rinsed in PBS, and incubated in 3% H_2_O_2_ to quench endogenous peroxidase activity. After washing with PBS, sections were incubated in 10% goat serum followed by 0.3% TritonX‐100 in PBS, then subsequently by overnight incubation at 4°C with anti‐TH antibody (T2928; Sigma–Aldrich, 1:1,000) in a humidified chamber. Immunoreactivity was detected with a biotinylated secondary antibody (PV‐9002 and ZLI‐9019; Zhongshan Golden Bridge Biotechnology Co., Ltd., Beijing, China) and diaminobenzidine.

The number of TH^+^ neurons in each section was counted via unbiased stereology with Stereo Investigator software (MBF Biosciences, Williston, VT, USA). Eight sections from each brain (every sixth section, 40 µm) were selected throughout the entire rostrocaudal extent of the substantia nigra pars compacta (SNpc), for examination. Using the optical fractionator principle, the SNpc was outlined on each section at 5× magnification, and TH^+^ neurons on the left sides were separately counted at 20× magnification using a brightfield microscope (Olympus, Tokyo, Japan). The weighted section thickness was used to correct for variations in tissue thickness at different sites.

The optical density (OD) of TH^+^ neurites in the striatum was determined using ImageJ software. The left striatum was selected as the measurement area. The OD of this area was measured and corrected by subtracting the nonspecific background signal. The average OD (OD/area) value was calculated.

### 4.19 Immunofluorescence (IF) Analysis and Confocal Microscopy

For animal experiments, the substantia nigra sections (25 µm) were rinsed in PBS and permeabilized with 0.3% Triton X‐100 in PBS for 10 min at room temperature. For cell experiments, Cell Climbing Tablet (YA0350; Solarbio Science & Technology Co., Ltd., Beijing, China) cells were fixed with 4% paraformaldehyde (PFA) for 30 min, washed with PBS, and permeabilized with 0.3% Triton X‐100 for 10 min. After blocking with 10% normal goat serum for 1 h, sections or cells were incubated overnight at 4°C with primary antibodies (Table S3) followed by Alexa Fluor 647‐, 594‐ or 488‐conjugated secondary antibody (A21244, A11032, A11037, A11076, A11034, and A11029; Thermo Fisher Scientific; 1:500) for 1 h at room temperature. Cell nuclei were visualized by counterstaining with 4′,6‐diamidino‐2‐phenylindole (DAPI) (D9542; Sigma–Aldrich). Sections were mounted on slides with aqueous mounting medium (F4680; Sigma–Aldrich), covered with coverslips, and visualized using a confocal microscope (880; Zeiss, Oberkochen, Germany). The IF intensity was quantified using ImageJ software.

### 4.20 Luminex Assay

Twelve cytokines (IFN‐γ, IL‐12p70, IL‐13, IL‐1β, IL‐2, IL‐4, IL‐5, IL‐6, TNF‐α, GM‐CSF, IL‐18, and IL‐10) in mouse cell culture supernatants and cell lysates were measured using the ProcartaPlex Mouse Th1/Th2 Cytokine Panel 11plex and ProcartaPlex Mouse IL‐10 Simplex (EPX110‐20820‐901 and EPX01A‐20614‐901, Thermo Fisher Scientific). The assay was performed and analyzed independently by Laizee Biotech (Shanghai, China) via the Luminex200 instrument and ProcartaPlex Analyst 1.0 software.

### 4.21 RNA Isolation and RT‐qPCR

The mRNA levels of IL‑1β, IL‑6, and TNF‑α were measured by RT‑qPCR. Total RNA was extracted from cells using FreeZol reagent (R711; Vazyme, Nanjing, China) according to the protocol of the manufacturer. Samples were centrifuged at 12 000 × *g* for 15 min at 4°C. RNAs in the aqueous phase were transferred to a clean tube and precipitated by the addition of isopropyl alcohol and centrifugation at 12 000 × *g* for 10 min at 4°C. The supernatant was removed, and the RNA pellet was washed once with 75% ethanol, air‑dried for 5–10 min, and dissolved in RNase‑free water. Total RNA was reverse‑transcribed into cDNA using the FastKing RT Kit (KR116; TIANGEN BIOTECH, Beijing, China). RT‑qPCR was performed in triplicate using the QuantStudio 5 Real‑Time PCR system (Thermo Fisher Scientific) with the SYBR Green qPCR kit (R0202; LABLEAD, Beijing, China). *Tab2* primers are listed in Table S4, and other inflammatory cytokine primer sequences are provided in our previous study [56]. The GAPDH housekeeping gene was used as an internal control to normalize target gene expression. Target gene expression was calculated relative to the internal control, and analysis was performed using the formula 2^−ΔΔCT^. The mean CT of triplicate measures was calculated for each sample.

### 4.22 ChIP‐qPCR

ChIP analysis was carried out with anti‐STAT3 antibodies (10253‐2‐AP; Proteintech, Rosemont, IL, USA) according to the manufacturer's instructions for the SimpleChIP Enzymatic Chromatin IP Kit (Magnetic Beads) (9003; Cell Signaling Technology). Querying the ChIP‐Atlas database identified a STAT3 binding region upstream of the *Tab2* 5′ untranslated region (5′UTR), and primers targeting this region were designed accordingly (Figure 4E). The primer sequences for the *Tab2* promoter were listed in Table S5. Fold enrichment was determined by qPCR and expressed as a percentage of input chromatin.

### 4.23 In Silico Structural Prediction and Virtual Screening

To predict the three‐dimensional structure of TAB2 and its interaction with α‐Syn, we employed the Protenix platform (v0.4.6) using FASTA‐formatted amino acid sequences of TAB2 and α‐Syn as input, with default parameters applied throughout the prediction pipeline [19]. The algorithm generated a ranked ensemble of five models. Based on subsequent experimental validation of K63‐linked polyubiquitin recognition capacity, Model 0 was deemed inadequate for representing TAB2; therefore, Model 4 (pTM = 0.78) was selected as the final structure for TAB2. For the TAB2‐α‐Syn complex, Model 0 exhibited the highest confidence metrics (pTM = 0.88, ipTM = 0.91, ΔiG = −38.9 kcal/mol) and was thus chosen for presentation.

Structure‐based virtual screening was performed using the MTiOpenScreen webserver (https://bioserv.rpbs.univ‐paris‐diderot.fr/services/MTiOpenScreen/) [20] and the Drug‐Rep webserver (http://cao.labshare.cn:10180/DrugRep/php) [21]. Molecular docking was executed separately against all five Protenix‐predicted models. Blind docking against the Drug‐lib database using MTiOpenScreen yielded extensive overlapping hits across models, whereas site‐directed docking targeting the NZF domain pocket using the Drug‐Rep Approved Drug Library produced non‐overlapping results. Consequently, we intersected the consensus outputs from MTiOpenScreen with Drug‐Rep predictions, identifying three common compounds for subsequent experimental validation.

### 4.24 SPR Assay

SPR binding assays were performed on a Biacore 8K system (Cytiva) to evaluate the binding affinity of lumacaftor for the TAB2‐NZF domain. The TAB2‐NZF domain was covalently immobilized on a CM5 sensor chip via amine coupling. Serial dilutions of lumacaftor in running buffer were injected over the chip surface. Real‐time sensorgrams were collected and double‐referenced against a blank/reference channel to correct for non‐specific binding and bulk refractive index changes. Kinetic and affinity parameters were calculated by fitting the data to a global 1:1 Langmuir binding model. The sensor surface was regenerated with glycine‐HCl between binding cycles.

### 4.25 Statistical Analysis

All statistical analyses were performed using GraphPad Prism version 10.6 (GraphPad Software, La Jolla, CA, USA). Comparisons between two groups were conducted using an unpaired two‐tailed t‐test. For multiple group comparisons involving one independent variable, one‐way ANOVA followed by Tukey's multiple comparisons test was used. Two‐way ANOVA was performed to evaluate the effects of two independent variables and their interaction. Data are presented as the mean ± SEM from at least three independent experiments. Differences were considered statistically significant at ^*^ *p* < 0.05, ^**^ *p* < 0.01, and ^***^ *p* < 0.001.

## Author Contributions

**Yanhao Zhao**: conceptualization, methodology, investigation, visualization, writing – original draft, writing – review and editing. **Zimeng Huang**: investigation, visualization. **Jianing Gao**: investigation. **Aolan Li**: investigation. **Huizhi Wang**: investigation. **Yingchuan Chen**: supervision. **Guanyu Zhu**: supervision. **Xin Zhang**: supervision. **Yin Jiang**: supervision. **Moxuan Zhang**: conceptualization, methodology, visualization, writing – original draft, writing – review and editing. **Fangang Meng**: supervision, writing – review and editing. **Jianguo Zhang**: supervision, writing – review and editing. **Tingting Du**: conceptualization, methodology, investigation, supervision, writing – original draft, writing – review and editing.

## Funding

Beijing Neurosurgical Institute Major Frontier Science and Technology Fund (No. 5), National Natural Science Foundation of China (No. 82101514), Beijing Natural Science Foundation Support Project (No. 7254306), Beijing High‐level Innovative and Entrepreneurial Talent Support Program (No. G202512038).

## Conflicts of Interest

The authors declare no conflicts of interest.

## Supporting information




**Supporting file**: advs77175‐sup‐0001‐SuppMat.docx

## Data Availability

The data that support the findings of this study are openly available in ModelArchive at https://modelarchive.org, reference number 10.5452/ma‐tab2‐pd.
