# Supplementary material for: TAB2 Causes Neuronal Damage by Aggravating Microglia‐Mediated Neuroinflammation in Parkinson's Disease
Source: Adv Sci (Weinh). 2026 Aug 17;13(62):e77175. doi: 10.1002/advs.77175 (PMC13479592; doi:10.1002/advs.77175)
Supplement: Supplementary file 1 [file ADVS-13-e77175-s001.docx]

Supporting Information

TAB2 Causes Neuronal Damage by Aggravating Microglia-Mediated Neuroinflammation in Parkinson’s Disease

Yanhao Zhao, Zimeng Huang, Jianing Gao, Aolan Li, Huizhi Wang, Yingchuan Chen, Guanyu Zhu, Xin Zhang, Yin Jiang, Moxuan Zhang*, Fangang Meng*, Jianguo Zhang*, Tingting Du*

**Figure S1. Human substantia nigra single-nucleus RNA sequencing (snRNA-seq) landscape in Parkinson’s Disease (PD).** (A, B) The Uniform Manifold Approximation and Projection (UMAP) plot showed the single-nucleus clustering data and subpopulation annotations from six healthy controls (HCs) and five PD patients in the substantia nigra, displayed separately by cluster number and patient. (C) UMAP feature plots showing canonical marker gene expression used for cell-type annotation. (D) The proportion of different cell types in each patient sample. (E) Box plot showing increased microglial abundance in PD patients. (F) The Gene Ontology enrichment analyses of differentially expressed pathways in PD microglia versus HCs.

**Figure S2. Cell-cell communication analysis and microglial subcluster characterization in PD.** (A) Circle plots showing differential cell-cell communication between PD and HCs, including the differential number of interactions and differential interaction strength. (B) Heat maps summarizing changes in intercellular communication, including differential interaction counts (left) and differential interaction strength (right) across sender-receiver cell- type pairs. (C, D) The UMAP plot showed the projections of microglia and displayed separately by patient and group. (E) Trajectory analysis showing the pseudotime progression of microglial subclusters. (F) Dot plot showing the expression patterns of representative marker genes across microglial subclusters. The size of the dot represents the percentage of cells expressing that gene in each cluster, and the color represents the average expression level. (G) Comparison of *TAB2* expression in MC1, MC2, and MC3 microglial subclusters between PD and HCs.

**Figure S3. High-dimensional weighted gene co-expression network analysis (hdWGCNA) network construction, co-expression modules and bulk validation of key genes.** (A) Selection of the soft-thresholding power for hdWGCNA in microglia, showing the scale-free topology model fit and mean connectivity across candidate powers. (B) Microglial gene dendrogram and identified co-expression modules based on hdWGCNA. (C) UMAP feature plots showing the module activity distribution for Microglia-M1 to Microglia-M8. (D–G) Box plots showing the expression differences of *AFF1*, *SORL1*, *INPP5D* and *TAB2* between PD and HCs in the substantia nigra bulk transcriptomic dataset.

**Figure S4. MAPK pathway activity at early time points following stimulation.** (A, B) Expression of MAPK pathway (p-ERK1/2, ERK1/2, p-p38, and p38) proteins in BV-2 cells treated with 0.5 mM MPP^+^ for 1, 2, 4, and 6 h (n = 3). (C, D) Expression of MAPK pathway (p-ERK1/2, ERK1/2, p-p38, and p38) proteins in BV-2 cells treated with 0.5 mM MPP^+^ for 5, 15, and 30 min (n = 3). Quantitative data are presented as the mean ± SEM. **P* < 0.05, ***P* < 0.01, and ****P* < 0.001.

**Figure S5. Experimental design and AAV-sh*Tab2* screening.** (A) Schematic timeline of AAV injection and experimental procedures. (B, C) Screening and selection of effective AAV-sh*Tab2* constructs (n = 4). (D–F) Validation of AAV transduction efficiency in microglia (Scale bar, 200μm; 50 μm) (n = 3). (G, H) Colocalization of TAB2 with Iba1 and TH in the substantia nigra of mice treated by AAV-sh*Tab2* (n = 3) (Scale bar, 20 μm). Quantitative data are presented as the mean ± SEM. **P* < 0.05, ***P* < 0.01, and ****P* < 0.001.

**Figure S6. Effects of rimegepant and paliperidone on cell viability and TAB2 expression after rimegepant, lumacaftor, and paliperidone treatments.** (A, B) CCK-8 assay results showing the dose-dependent effect of rimegepant and paliperidone on BV-2 cell viability. The IC50 values were determined to be 32.37 μM for rimegepant and 116.6 μM for paliperidone. (C–G) TAB2 expression in BV-2 cells treated with rimegepant, lumacaftor or paliperidone for 36 hours with different concentrations (n = 6). Quantitative data are presented as the mean ± SEM. **P* < 0.05, ***P* < 0.01, and ****P* < 0.001.

**Figure S7. *In vivo* dose screening of lumacaftor.** (A–D) Dose-dependent effects of lumacaftor on TAB2 expression in the substantia nigra (A, B) and striatum (C, D) of mice after intraperitoneal administration (n = 3). Quantitative data are presented as the mean ± SEM. **P* < 0.05, ***P* < 0.01, and ****P* < 0.001.

**Figure S8. Experimental design and lumacaftor inhibited TAB2-mediated NF-κB pathway activation and exerted neuroprotective effects in the striatum of PD mouse models.** (A) Schematic timeline of lumacaftor injection and experimental procedures. (B–E) Expression of TAB2-related (TAB2, p-TAK1, TAK1), PD-related (TH, α-synuclein), NF-κB pathway (p-p65, p65, p-IκBα, and IκBα), and apoptosis pathway (Bcl-2, BAX, Caspase-3, and cleaved Caspase-3) proteins in the striatum of 1-methyl-4-phenyl-1,2,3,6-tetrahydropyridine mouse models followed by lumacaftor pretreatment (n = 3). (F, G) Colocalization of TAB2 with Iba1 and TH in the substantia nigra of mice treated by lumacaftor (n = 3) (Scale bar, 20 μm). Quantitative data are presented as the mean ± SEM. **P* < 0.05, ***P* < 0.01, and ****P* < 0.001.

**Table S1. siRNA sequences**

| **No.** | **Target sequence (5′ to 3′)** |
| --- | --- |
| si*Tab2*-1 | GCTGCCAGTCCTCCAAATA |
| si*Tab2*-2 | GCACATGTGGATAGAATAA |
| si*Tab2*-3 | GCAGTTGAGAAGTTGTAAT |

**Table S2. AAV-sh*Tab2* sequences**

| **No.** | **Target sequence (5′ to 3′)** |
| --- | --- |
| AAV-sh*Tab2*-1 | GCATCTTGGATTTCACCTAGG |
| AAV-sh*Tab2*-2 | GCACTTCCTCATTGGTCAACA |
| AAV-sh*Tab2*-3 | GCTCTAGCACATGTGGATAGA |

**Table S3. Primary antibody for western blot and immunofluorescence.**

| Antibody | Dilution ratio | Catalog No. |
| --- | --- | --- |
| TAB2 | 1:1,000 | 14410-1-AP, Proteintech, Rosemont, IL, USA |
| α-Synuclein | 1:1,000 | 4179S, Cell Signaling Technology, Danvers, MA, USA |
| TH | 1:2,000 | T2928, Sigma-Aldrich, St. Louis, MO, USA |
| K63-Ub | 1:1,000 | 5621S, Cell Signaling Technology, Danvers, MA, USA |
| p-TAK1 | 1:1,000 | 4508S, Cell Signaling Technology, Danvers, MA, USA |
| TAK1 | 1:1,000 | 12330-2-AP, Proteintech, Rosemont, IL, USA |
| p-p65 | 1:1,000 | 3033S, Cell Signaling Technology, Danvers, MA, USA |
| p65 | 1:1,000 | 8242S, Cell Signaling Technology, Danvers, MA, USA |
| p-IκB | 1:1,000 | 2859S, Cell Signaling Technology, Danvers, MA, USA |
| IκB | 1:1,000 | 4812S, Cell Signaling Technology, Danvers, MA, USA |
| p-ERK | 1:1,000 | 4370S, Cell Signaling Technology, Danvers, MA, USA |
| ERK | 1:1,000 | 4695S, Cell Signaling Technology, Danvers, MA, USA |
| p-p38 | 1:500 | sc-166182, Santa Cruz Biotechnology, Dallas, TX, USA |
| p38 | 1:1,000 | 14064-1-AP, Proteintech, Rosemont, IL, USA |
| p-STAT3 | 1:1,000 | ET1603-40, Huabio, Hangzhou, China |
| STAT3 | 1:2,000 | 10253-2-AP, Proteintech, Rosemont, IL, USA |
| GST | 1:3,000 | 10000-0-AP, Proteintech, Rosemont, IL, USA |
| Cleaved Caspase-3 | 1:1,000 | 9661S, Cell Signaling Technology, Danvers, MA, USA |
| Caspase-3 | 1:1,000 | 9662S, Cell Signaling Technology, Danvers, MA, USA |
| BAX | 1:20,000 | 50599-2-Ig, Proteintech, Rosemont, IL, USA, |
| Bcl-2 | 1:5,000 | 68103-1-Ig, Proteintech, Rosemont, IL, USA |
| IL-1R1 | 1:1,000 | 27348-1-AP, Proteintech, Rosemont, IL, USA |
| β-actin | 1:10,000 | 81115-1-RR, Proteintech, Rosemont, IL, USA |
| GAPDH | 1:10,000 | 60004-1-Ig, Proteintech, Rosemont, IL, USA |
| TH (for IF) | 1:1,000 | T2928, Sigma-Aldrich, St. Louis, MO, USA |
| Iba1 (for IF) | 1:1,000 | 019-19741, FUJIFILM Wako Pure Chemical Corporation, Osaka, Japan |
| TAB2 (for IF) | 1:20 | 14410-1-AP, Proteintech, Rosemont, IL, USA |
| Iba1 (for IF) | 1:600 | OB-PGP049-01, Oasis Biofarm, Hangzhou, China |
| iNOS (for IF) | 1:50 | sc-7271, Santa Cruz Biotechnology, Dallas, TX, USA |
| CD206 (for IF) | 1:100 | HA601453, Huabio, Hangzhou, China |

**Table S4. *Tab2* primer sequences**

| **Primer** | **Target sequence (5′ to 3′)** |
| --- | --- |
| *Tab2*-F | CTTGGACTTGCAGTCACAGA |
| *Tab2*-R | CTGTCTGTGGCTCTTGCTGA |

**Table S5. ChIP-qPCR *Tab2* promoter primer sequences**

| **Primer** | **Target sequence (5′ to 3′)** |
| --- | --- |
| ChIP-*Tab2*p-F | GGCTCCTCTCTCTCTCCCTC |
| ChIP-*Tab2*p-R | AAACACCGCGAGATCTGAGG |
